# Rational Design of Profile HMMs for Sensitive and Specific Sequence Detection with Case Studies Applied to Viruses, Bacteriophages, and Casposons

**DOI:** 10.3390/v15020519

**Published:** 2023-02-13

**Authors:** Liliane S. Oliveira, Alejandro Reyes, Bas E. Dutilh, Arthur Gruber

**Affiliations:** 1 Department of Parasitology, Instituto de Ciências Biomédicas, Universidade de São Paulo, São Paulo 05508-000, SP, Brazil; 2 Max Planck Tandem Group in Computational Biology, Department of Biological Sciences, Universidad de los Andes, Bogotá 111711, Colombia; 3 The Edison Family Center for Genome Sciences and Systems Biology, Washington University School of Medicine, Saint Louis, MO 63108, USA; 4 Institute of Biodiversity, Faculty of Biological Sciences, Cluster of Excellence Balance of the Microverse, Friedrich-Schiller-University Jena, 07743 Jena, Germany; 5 Theoretical Biology and Bioinformatics, Science for Life, Utrecht University, Padualaan 8, 3584 CH Utrecht, The Netherlands; 6 European Virus Bioinformatics Center, Leutragraben 1, 07743 Jena, Germany

**Keywords:** viral classification, viral detection, profile HMMs, sequence classes, mutual information theory, sequence entropy

## Abstract

Profile hidden Markov models (HMMs) are a powerful way of modeling biological sequence diversity and constitute a very sensitive approach to detecting divergent sequences. Here, we report the development of protocols for the rational design of profile HMMs. These methods were implemented on TABAJARA, a program that can be used to either detect all biological sequences of a group or discriminate specific groups of sequences. By calculating position-specific information scores along a multiple sequence alignment, TABAJARA automatically identifies the most informative sequence motifs and uses them to construct profile HMMs. As a proof-of-principle, we applied TABAJARA to generate profile HMMs for the detection and classification of two viral groups presenting different evolutionary rates: bacteriophages of the *Microviridae* family and viruses of the *Flavivirus* genus. We obtained conserved models for the generic detection of any *Microviridae* or *Flavivirus* sequence, and profile HMMs that can specifically discriminate *Microviridae* subfamilies or *Flavivirus* species. In another application, we constructed Cas1 endonuclease-derived profile HMMs that can discriminate CRISPRs and casposons, two evolutionarily related transposable elements. We believe that the protocols described here, and implemented on TABAJARA, constitute a generic toolbox for generating profile HMMs for the highly sensitive and specific detection of sequence classes.

## 1. Introduction

A multiple sequence alignment (MSA) contains information about the evolutionary history of a group of homologous proteins. According to the neutral model of evolution [1], most deleterious mutations are removed by negative selection, while those that are neutral may be kept [2]. Since the level of residue conservation is heterogenous in protein sequences, the most conserved regions are those that present structural and functional constraints and, therefore, show low substitution rates. Conversely, sequence regions that are more tolerant to variations may accumulate neutral mutations and display high substitution rates. While conserved domains show a high density of sites that are pivotal to protein function, and are conserved across multiple orthologs (synapomorphies), they also contain residues that are distinctive to orthologs of a specific taxon or group of taxa (autapomorphies). These residues may confer specific roles for a subfamily of proteins or represent neutral mutations that became fixed in their specific lineage.

Highly conserved regions detected in MSAs may, therefore, be indicative of common secondary structures, active functional sites, substrate binding sites, among other features [3,4,5,6,7]. Several methodological approaches have been developed to assign conservation scores on MSAs [2,8,9]. One of the most effective methods [10] that implemented an algorithm based on Jensen–Shannon divergence (JSD) [11] assumes that amino acid distributions show distinct patterns between conserved sites and positions under no evolutionary pressure. The implementation considered the estimated conservation of sequentially neighboring sites and identified functionally important residues significantly better than other protein conservation metrics. This result was corroborated by another study [12] in which frequency-based metrics that consider background distribution, such as JSD, were the most effective at predicting catalytic sites in the alignments.

Another important application of an MSA is the identification of autapomorphic sites that can discriminate paralogs with specialized protein functionalities or sets of sequences belonging to distinct taxonomic groups. Several methods have been proposed for such application, using sequence entropy [5,13,14], mutual information [9,15], and a combination of mutual information and statistical methods [3,16,17,18].

MSAs also established the ground for the development of sensitive tools to classify protein families using protein sequence profiles [19], Position-Specific Score Matrices (PSSMs) [20] and profile hidden Markov models (profile HMMs) [21,22]. In addition, large collections of such profiles have become publicly available, including the CDD (Conserved Domain Database) [23] for PSSMs and Pfam (Protein Families database) [24] for profile HMMs. These model collections have been largely used to detect remote homologs of evolutionary distant organisms and to ascribe biological functions to novel sequences.

Homology-based identification and classification of viral sequences in genomic and metagenomic data is usually performed with pairwise similarity searches of their assembled sequences against annotated reference databases [25,26,27]. However, it has been demonstrated that pairwise comparisons only detect one-half of the relationships between proteins with 20–30% identity [28]. In the case of viral sequences, this is a very common scenario, since viruses present high mutation rates, especially RNA viruses [29,30,31,32,33]. In addition to the high level of divergence observed in viruses, viral sequence diversity has still been scarcely sampled compared to prokaryotes [34]. The low representation of many viral families, together with the relatively low sensitivity of pairwise comparisons, results in many metagenomics studies identifying and classifying only a relatively small fraction of the viral sequences present in metagenomic samples [35]. The growing pace of viral genome sequencing through metagenomics, makes the proper identification and classification of novel viruses even a more challenging task [36,37]. The scarcity of viral sequences is being changed with the advent of new public resources such as the IMG/VR, which now comprises viral genome sequences of cultivated and uncultivated viruses [38,39].

Profile-based similarity search methods, which can aggregate and quantify variations within a sequence dataset, represent a more sensitive approach than sequence-based pairwise similarity searches. In fact, for sequences showing less than 30% identity, profile-based methods are capable of detecting up to three times more sequences than pairwise alignments [40]. Profile HMMs [22,41] are linear probabilistic models constructed from MSAs that incorporate the full diversity of residues from each alignment position, including insertion and deletion events (indels). Similarity searches using profile HMMs can be performed with different software packages such as HMMER [21] for searching profile HMMs versus biological sequences and HH-suite3 for profile-to-profile searches [42].

Unlike prokaryotic and eukaryotic organisms that share genes that can be used as universal markers (e.g., the small subunit ribosomal RNA and cytochrome b), viral genomes are derived from multiple evolutionary origins, with some viral groups being clearly polyphyletic [43], which result in a much higher diversity than cellular organisms. Thus, at the best scenario, viral molecular markers can only be used as taxon signatures of restricted groups of viruses. Given the high recombination rates across distinct viral genomes and their modular character, proteins may contain domains with different taxonomic specificities, with some being restricted to narrow taxonomic groups of viruses, while others showing conservation across wider groups. Hence, using full-length protein sequences or profile HMMs derived from such sequences as taxonomic markers is a very challenging task. A possible way to overcome this situation is to identify protein regions that are rich in conserved and discriminative amino acid positions. Since MSAs represent a portrait of sequence diversity of homologous proteins derived from distinct taxa, they represent a solid foundation on which to unravel such taxon-specific stretches.

Profile HMMs have been increasingly used in virus studies, including astrovirus [44], Influenza A viruses [45,46], *Microviridae* phages [47], coronaviruses [48], mitovirus [49], herpesviruses [49,50], and African swine fever virus (ASFV) [51]. Additionally, profile HMMs can be used as seeds to nucleate sequencing reads in progressive assemblies for a targeted specific reconstruction of viral sequences from metagenomic datasets [34,47,52]. Several different databases of viral profile HMMs have been developed and are publicly available, such as pVOGs [53] for prokaryotic viruses and vFam for eukaryotic viruses [54]. However, these databases have not been updated in recent years and present some limitations, such as the highly biased representation of the different viral families and the low number of sequences used to build most of the models [34]. Some recently developed resources include RVDB-prot [55], a protein version of the Reference Viral DataBase (RVDB) [56], viralOGs/eggNOG v5.0 [57], efam [58], PHROGs [59], ViPhOGs [60,61], Cenote-Taker 2 hallmark gene HMM database [62], and IMG/VR [39], a database of viral genome sequences of cultivated and uncultivated viruses that includes thousands of profile HMMs. Most if not all available viral databases provide profile HMMs derived from MSAs containing orthologs, that is, sequences that are assumed to share a common ancestry and biological function. Such approach implies that some models are built from sequences belonging to diverse taxa and, as such, are much more suitable for finding other orthologs than to be used for the detection and classification of sequences into specific taxa.

No tool is currently available for determining positions that are either conserved across all sequences of an MSA or restricted to a specific subgroup of sequences. Moreover, none of these methods determine regions that are rich in synapomorphic and autapomorphic sites. This situation stimulated us to carry out different tests using arbitrary regions and then develop and implement TABAJARA, a program that integrates a variety of methods to find conserved and specific regions in nucleotide and protein sequences. This tool constructs profile HMMs capable of detecting sequences that share conserved regions or, alternatively, discriminate groups of sequences defined by subtype-specific functional sites and/or taxonomic criteria. Here, we describe the concepts implemented on TABAJARA and present several study cases. Among possible applications, we cover prokaryotic and eukaryotic viruses presenting different levels of divergence, and casposons, a superfamily of transposable elements [63]. Finally, we discuss other potential applications of the methodology to other biological domains.

## 2. Materials and Methods

### 2.1. Data Sources

#### 2.1.1. *Microviridae*

A dataset composed of 81 sequences of the major capsid protein (VP1) from different subfamilies of the *Microviridae* family, as described by Roux et al. [64], was obtained from http://dx.doi.org/10.5061/dryad.8ht80 (URL accessed on 2 February 2023), plus two sequences of accession numbers YP_004732986 and NP_044312. This dataset comprises 33 sequences of the *Alpavirinae* subfamily, 44 of *Gokushovirinae*, and 6 of *Pichovirinae*. Another dataset of *Microviridae*, kindly provided by Prof François Enault (Université Clermont Auvergne, Clermont-Ferrand, France), consisted of 1866 VP1 sequences comprising 501 from *Alpavirinae*, 1040 from *Gokushovirinae*, and 325 from *Pichovirinae*. Since the original dataset from Roux et al. [64] contained only six *Pichovirinae* sequences, we incremented it by adding 30 more sequences of this subfamily from the 1866-sequence dataset, resulting in a final 113-sequence dataset that was used for training purposes. The 1866-sequence dataset, depleted from the 30 *Pichovirinae* sequences, constituted a final test set composed of 1836 sequences, with an average sequence length of 567 aa residues.

#### 2.1.2. *Flavivirus*

A total of 127 polyprotein sequences covering species diversity of the genus *Flavivirus* were manually selected and downloaded from the NCBI’s protein database. This dataset, comprising 20 sequences of the four types of *Dengue virus* (DENV-1 to DENV-4), 10 of Zika virus (ZIKV), and 10 of Yellow fever virus (YFV), plus 87 sequences from 63 other *Flavivirus* species, was used as a training set. A more comprehensive dataset, downloaded from the same source, comprised polyprotein sequences of all available flaviviruses, excluding the 127 sequences used for the training set. Incomplete and/or identical sequences were discarded, as well as those containing more than two ambiguities. The final dataset, used as a test set, was composed of 6364 sequences, including 3919 of DENV, 327 of ZIKV, 63 of YFV, and the remaining 2055 sequences covering all other available flaviviruses. The average protein sequence length of this dataset is 3407 aa residues.

#### 2.1.3. Casposons

We used a dataset of endonuclease Cas1 protein sequences from bona fide casposons and CRISPR elements. This dataset is composed of 54 sequences covering the four casposon families [65] and 52 selected representatives of CRISPR-Cas systems [66].

### 2.2. Multiple Sequence Alignments

Protein sequence datasets were aligned using the MUSCLE [67] program with default parameters and the resulting alignment files, used throughout this work, are available in the Supplementary Material.

### 2.3. Profile HMM Construction

Profile HMMs were generated using MSA files as input and the program hmmbuild (HMMER v3.3 package) [21] with default parameters.

### 2.4. Similarity Searches

Similarity searches were performed using profile HMMs as queries against different protein datasets with the hmmsearch program [21]. Searches were executed using either default parameters or, alternatively, employing cutoff scores specified by the parameter -T of the program.

### 2.5. Implementation of TABAJARA Program

TABAJARA (Tool for Alignment Block Analysis Joining Appropriate Rational Approaches) is a program for the rational design of profile HMMs that uses MSA files as input (FASTA or CLUSTAL format). The program automatically detects nucleic acid or protein sequence data. IUPAC nucleotide ambiguity characters, and the X character for amino acid ambiguity are ignored. TABAJARA can produce models from full-length sequence MSAs or, alternatively, generate short profile HMMs from the most informative blocks of the alignment. To identify these blocks, TABAJARA uses position-specific information scores ascribed to all columns of the alignment, according to different metrics. Two execution modes are available to the user: (1) conservation, to generate models capable of detecting all sequences of the MSA; and (2) discrimination, to construct models that are discriminative for two specific groups of sequences. TABAJARA was written in Perl (http://www.perl.org—URL accessed on 2 February 2023) and uses the programs hmmsearch, hmmbuild and nhmmer from HMMER package [21] and MUSCLE [67]. The program, usage manual, tutorials, and suggested configuration files are available at https://github.com/gruberlab/TABAJARA (URL accessed on 2 February 2023).

### 2.6. Position-Specific Scoring Metrics

As a first step towards identifying distinctive regions, TABAJARA calculates position-specific scores, ranging from 0 to 1 along all columns of an MSA. Alignment columns presenting a proportion of gap characters above a user-defined threshold are scored with a zero value. For the remaining columns, a variety of metrics can be used, as explained below, depending on the type of input sequence (nucleotide or protein) and execution mode.

#### 2.6.1. Jensen–Shannon Distance

To determine the level of character conservation across all protein sequences of an MSA, we implemented a previously described algorithm [10], based on the Jensen–Shannon divergence method [11]. For a column *c*, Jensen–Shannon divergence $D_{c}^{JS}$ is defined as

(1) $$D_{c}^{JS}=\lambda RE_{P_{c},r}+\left(1-\lambda \right)RE_{\left(q,r\right)}$$

where *RE* (Kullback–Leibler divergence) is the relative entropy [9,10], $r=\lambda p_{C}+\left(1-\lambda \right)q$, $p_{c}$ is the alignment column amino acid distribution, *q* is a background distribution (we use the overall amino acid distribution in the BLOSUM62 alignment) and *λ* is a prior weight (with a value of ½).

#### 2.6.2. Shannon Entropy

In the case of nucleic acid data, TABAJARA calculates the Shannon entropy [68,69] $E_{c}$ at an alignment column *c* as:

(2) $$E_{c}=-\underset{m=\left\{A,C,G,T,-\right\}}{\sum }p_{c_{m}}log_{b}p_{c_{m}}$$

where $p_{c_{m}}$ is the probability of occurrence of a DNA base *m* and *b* is the base of the logarithm (we use base 2). To obtain a range of 0 to 1, entropy values are normalized assuming a minimum value of 0, when one state has a frequency equal to 1 (100%), and a maximum value $E_{max}$ of 2.32, when all possible states (A, C, G, T, −), including gaps, are equiprobable. Since we are interested to measure the level of conservation of each alignment position, TABAJARA calculates the final conservation score $CS_{c}$ at an alignment column *c* as

(3) $$CS_{c}=1-E_{c}/E_{max}$$

#### 2.6.3. Mutual Information

To measure the ability of alignment columns to discriminate two groups, TABAJARA uses mutual information (MI) [9,15] for both, DNA and protein sequence alignments. The MI value $I_{c}$ at an alignment column *c* is defined by

(4) $$I_{c}=\overset{2}{\underset{i=1}{\sum }}\overset{n}{\underset{\propto =1}{\sum }}p_{c}\left(\propto ,i\right)log\frac{p_{c}\left(\propto ,i\right)}{p_{c}\left(\propto \right)p_{c}\left(i\right)}$$

where *n* is 5 (4-character set of bases plus the gap symbol) or 21 (20-character set of amino acids plus the gap symbol), $p_{c}\left(\propto ,i\right)$ is the joint probability function of a character (DNA base or amino acid residue *α*) and a group *i*, whereas $p_{c}\left(\propto \right)$ and $p_{c}\left(i\right)$ are the marginal probability functions of a character *α* and a group *i*, respectively.

#### 2.6.4. Sequence Disharmony

An additional measure of the group discrimination ability of alignment positions is sequence disharmony (SD), that we developed based on the sequence harmony (SH) method [13,14]. SD generates values ranging from 0 for harmonious sequence groups to 1 for totally dissimilar groups. Given two groups A and B, the original definition of SD at an alignment column *c* is described by

(5) $${\mathrm{SD}}_{c}=1+\frac{1}{2}\left(E_{c}^{A+B}-E_{c}^{A}-E_{c}^{B}\right)$$

and

(6) $$E_{c}^{A+B}=-\sum \left(p_{A}+p_{B}\right)log\left(p_{A}+p_{B}\right)$$

where separate ($E_{c}$—Equation (2)) and combined ($E_{c}^{A+B}$—Equation (6)) entropies of each group are calculated.

### 2.7. Protein Similarity Calculation

To calculate the mean similarity across all proteins of specific taxonomic groups, we used the Needle program from the EMBOSS package [70]. Needle performs an optimal global pairwise alignment (including gaps) by implementing the Needleman–Wunsch algorithm. Thus, all combinations of protein pairs from each dataset were submitted to pairwise alignments with Needle using default parameters and BLOSUM62 as the substitution scoring matrix. For each dataset of protein sequences, we used all calculated similarity values to determine the respective arithmetic mean and standard deviation. Minimum and maximum similarity values were also stored.

### 2.8. K-Fold Cross-Validation

To assess generalization performance of TABAJARA, we constructed profile HMMs using k-fold cross-validations for *Flavivirus* and *Microviridae* protein sequence datasets. Datasets were randomly divided into 10 subsamples (*k* = 10), preserving in each subsample the proportion between the viral group being tested and the other sequences of the dataset. In the case of the genus *Flavivirus*, we tested DENV versus all other non-DENV. For *Microviridae* family, *Gokushovirinae* was tested against sequences of *Alpavirinae* and *Pichovirinae* subfamilies. The validation process was performed in *k* iterations, with a single subsample being selected as a test set and the remaining nine (*k*-1) used as a single training set in each iteration. Sequences of these nine subsamples were aligned with MUSCLE and the generated MSA was used as an input to run TABAJARA in discrimination mode for DENV, in the case of *Flavivirus*, and *Gokushovirinae* for *Microviridae*, respectively. All models were tested in similarity searches using the hmmsearch program against the sequences of the respective subsample selected as the test set of the iteration. For each iteration, sensitivity and specificity values were calculated for individual models and the corresponding arithmetic means of these values were determined.

## 3. Results

### 3.1. Full-Length Protein Sequences for Profile HMM Construction

**Figure 1:**
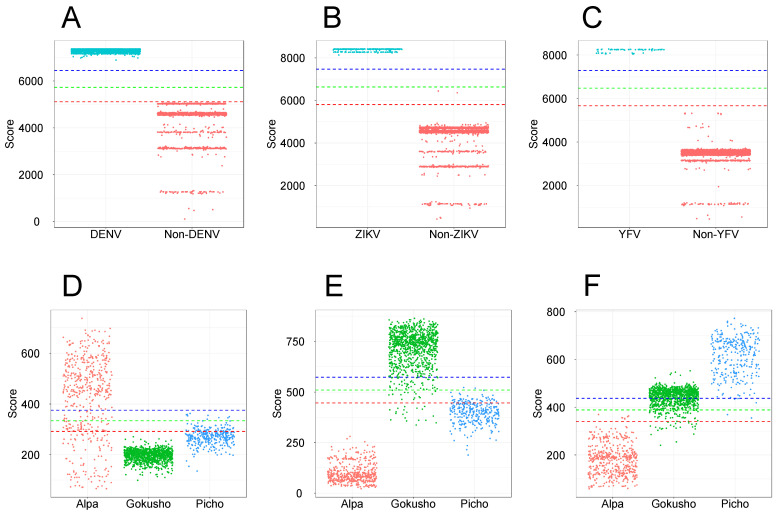
Full-length protein sequences as a source for the construction of discriminative profile HMMs. Profile HMMs of DENV (**A**), ZIKV (**B**) and YFV (**C**) were constructed and tested by similarity searches using a training set composed of specific polyprotein sequences and tested with hmmsearch program against a bona fide dataset of 6364 Flavivirus sequences comprising a wide variety of viral species. For *Microviridae*, profile HMMs were constructed using sequences of the major capsid protein (VP1) specific to *Alpavirinae* (**D**), *Gokushovirinae* (**E**), and *Pichovirinae* (**F**) subfamilies and the models were validated against a bona fide dataset of 1866 VP1 sequences of *Microviridae*, comprising members of three subfamilies. Each column scatter plot depicts the results obtained for the tested species/subfamily the remaining viruses of the group, according to the respective alignment scores. The dots correspond to scores obtained by each profile HMM against the different protein sequences of the tested datasets. Dashed horizontal lines indicate cutoff scores corresponding to 70% (red), 80% (green), and 90% (blue) of the alignment score observed for the lowest hit of the training set.

Most of the publicly available resources of viral profile HMMs [54,57,71] use full-length protein sequences to generate orthologous groups and profile HMMs are built from their respective MSAs. To evaluate the ability of full-length sequences to generate taxon-specific profile HMMs, we employed DENV and *Flavivirus* sequences as a case study. We used a training set composed of 20 DENV polyprotein sequences (derived from the 127-sequence dataset—see Section 2.1.2) to produce an MSA and build a profile HMM. This model was used as a query against the 20-sequence training set and the test set of 6364 *Flavivirus* sequences. All dengue sequences were detected, including the 20 sequences of the training set and 3919 dengue sequences (100% recall) of the test set (Figure 1A). Notably, 2445 non-dengue flaviruses were also detected, but with significantly lower alignment scores, opening the possibility that the use of appropriate score cutoff values could efficiently discriminate both groups. To address this point, we implemented an approach in which the model was used as a query against the 127-sequence *Flavivirus* dataset, and cutoff scores were set to 70, 80, and 90% of the alignment score observed for the last dengue hit in the training set. These cutoff score values were then compared to the scores obtained by the DENV model against the 6364-sequence dataset. As can be seen in Figure 1A, a value corresponding to 80% of the score could be used as a suitable cutoff score for the full-length sequence model. Using the same criterion for determination of cutoff scores, we obtained similar discriminative results for full-length models of ZIKV (Figure 1B) and YFV (Figure 1C).

We extended this test to sequences of the *Microviridae* family, which are much more diverse than the *Flavivirus* sequences. In this case, we built profile HMMs for *Alpavirinae*, *Gokushovirinae,* and *Pichovirinae* subfamilies using subsets of 33, 44, and 36 sequences of VP1, respectively, obtained from the *Microviridae* training set (see Section 2.1.1). Figure 1D–F show that the alignment scores observed for sequences of the *Microviridae* subfamilies revealed a highly heterogeneous range of scores for *Alpavirinae* (Figure 1D), which strongly overlapped with the alignment scores obtained for *Gokushovirinae* and *Pichovirinae* sequences, hampering the assignment of effective cutoff scores. In addition, profile HMMs constructed from full-length *Gokushovirinae* (Figure 1E) and *Pichovirinae* (Figure 1F) VP1 sequences also showed score overlapping, which resulted in cross-detection between these subfamilies.

Similar to what was observed for *Flavivirus*, taxon-specific models, derived from full-length protein sequences, showed recall values of 100% for the three tested *Microviridae* subfamilies. However, given the high rate of score overlap across the different subfamilies (Figure 1D–F), the specificity of the models was null for all subfamilies (Table S1). The use of score cutoff values (70, 80 and 90%) significantly improved specificity, but at the cost of sacrificing sensitivity, with no cutoff value displaying a good balance between sensitivity and specificity, especially for the more diverse *Alpavirinae* subfamily. Our conclusion is that full-length sequences can generate profile HMMs that are highly sensitive for the detection of wide taxonomic groups such as the *Flavivirus* genus and *Microviridae* family. On the other hand, models designed for lower taxonomic levels such as DENV, ZIKV, and YFV required the use of suitable cutoff scores to present a good discrimination (Figure 1A–C). In the case of *Microviridae*, where each subfamily presents a high diversity across its own members, divergent regions in the MSA may introduce significant noise to the corresponding profile HMMs, thus limiting their discrimination ability, with score overlaps being observed among the distinct subfamilies. In such a condition, which possibly applies to many other viral groups, the use of cutoff scores yields a substantial drop of recall values. In conclusion, profile HMMs derived from full-length sequences may represent valuable markers for some taxa, but this cannot be universally extended to all viral groups and their respective taxonomic levels, nor is it useful for screening unassembled data.

### 3.2. Short Alignment Blocks as a Source for the Construction of Profile HMMs

In the former section, we described the limitations of using profile HMMs derived from full-length protein sequences. Thus, we decided to develop a rational approach for profile HMM design, starting by evaluating whether short alignment regions could be able to generate models specific to a particular group of sequences (e.g., a viral species) and, if so, what would be the ideal length of such regions. For this evaluation, we generated a repertoire comprising all possible alignment blocks falling within a given size range and used the whole set of short blocks for the construction of discriminative profile HMMs. Analogously to the test performed for full-length profile HMMs, we used two study cases: three viral species (DENV, ZIKV, and YFV) of the *Flavivirus* genus and three subfamilies of the *Microviridae* family. In the case of flaviviruses, we used training sets composed of full-length polyprotein sequences of DENV, ZIKV, and YFV (derived from the 127-sequence dataset—see Data Sources section). The sequence datasets were aligned and sliding windows varying one position at a time, ranging from 10 to 110 amino acids, were used to delimit the alignment blocks. These blocks were then extracted and used to build profile HMMs. The same approach was extended to *Alpavirinae*, *Gokushovirinae,* and *Pichovirinae* subfamilies, using 33, 44, and 36 sequences of VP1, respectively, derived from the 113-sequence dataset (see Section 2.1.1). We used the test datasets of 6364 *Flavivirus* polyprotein sequences and 1836 *Microviridae* VP1 sequences to estimate the sensitivity and specificity of all constructed models.

**Figure 2:**
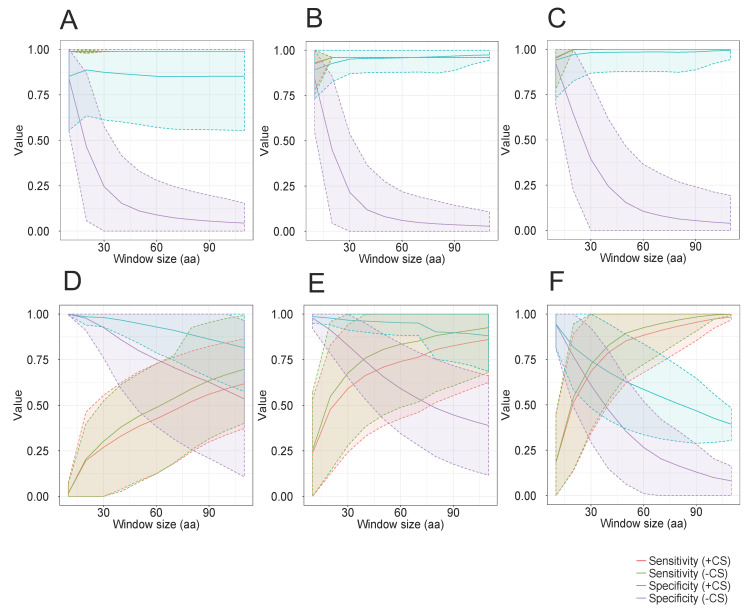
Evaluation of a complete repertoire of alignment blocks of variable size as candidates for the construction of discriminative profile HMMs. Sliding windows of different sizes, starting from a minimum length of 10 aa, and varying in increments of 10 aa each, were used throughout MSAs of full-length polyprotein sequences specific for DENV (**A**), ZIKV (**B**) and YFV (**C**) (*Flavivirus* genus) or alignments of major capsid protein (VP1) sequences specific to *Alpavirinae* (**D**), *Gokushovirinae* (**E**) and *Pichovirinae* (**F**) subfamilies (*Microviridae* family). Alignment blocks representing the entire repertoire obtained for each window size, were used to construct profile HMMs. All *Flavivirus*-derived models were tested with hmmsearch program against a bona fide dataset of 6364 *Flavivirus* sequences comprising a wide variety of viral species. For *Microviridae*, profile HMMs were validated against a bona fide dataset of 1836 VP1 sequences, comprising members of three subfamilies of this family. Sensitivity and specificity lines, using (+CS) or not (-CS) cutoff scores, represent arithmetic means calculated from the combined values of the multiple models generated for each alignment block size. Shaded areas indicate standard deviation values. Cutoff scores correspond to 80% of the alignment score observed for the lowest hit detected by each individual model against the corresponding training set.

In the case of DENV, ZIKV, and YFV (Figure 2A–C, respectively), models constructed from alignment blocks of 10 positions showed the best performance. For instance, we observed a sensitivity and specificity of 0.9 and 0.84, respectively, for DENV (Figure 2A). Similar results were also obtained for ZIKV (Figure 2B) and YFV (Figure 2C). Profile HMMs constructed from longer alignment blocks showed a very high sensitivity, but at the cost of a strong decrease in specificity. In the case of *Microviridae*, a similar drop of specificity was observed for the three analyzed subfamilies (Figure 2D–F, respectively). On the other hand, the observed sensitivity values were much lower than those obtained for flaviviruses and showed an increase when using models constructed from longer alignment blocks. These results can be ascribed to the fact that *Microviridae* phages of any subfamily are much more diverse than any viral species of the genus *Flavivirus*. Thus, longer models would in theory present a higher chance to cover sequence stretches that are conserved across all members of the viral taxon. Nevertheless, the very low specificity values obtained with long models make their use unfeasible for taxon discrimination purposes.

To improve the specificity of the longer profile HMMs, we implemented cutoffs in the alignment scores with a heuristic approach, using DENV as a proof-of-principle. First, all models were submitted to similarity searches against the 127-sequence dataset of *Flavivirus* polyproteins. For profile HMMs identifying only DENV sequences, the cutoff was set at 80% of the alignment score of the lowest-scoring hit. For profile HMMs that detected a mixture of dengue and non-dengue sequences, we screened the results in decreasing order of score until the first hit of a non-dengue sequence was found. The cutoff score was then calculated as being 80% of the score observed for the immediately preceding dengue hit. Using these assigned cutoff scores, we repeated the evaluation of the models derived from the entire repertoire of short alignment blocks. We observed a huge improvement in the specificity of DENV detection (Figure 2A), indicating that even longer alignment blocks can be used to produce taxon-specific models, provided that proper cutoff scores are used. Similar results were also observed for models constructed from sequences of ZIKV (Figure 2B) and YFV (Figure 2C). For *Microviridae* (Figure 2D–F), the use of cutoff scores produced a noticeable improvement in the specificity in longer models, but not as remarkable as that observed in flaviviruses. Additionally, there was a slight decrease in the recall rate in the three tested subfamilies. This body of evidence suggests that short models can potentially be used for the detection of closely related groups of sequences, with high recall and specificity values. For more distantly related sequences, short models show a low recall, whereas longer models present an unacceptably low specificity. Finally, the use of cutoff scores may represent a valuable improvement of the method, but they are definitely not sufficient to allow the general use of unselected short alignment blocks for the construction of taxon-specific profile HMMs.

### 3.3. A Rational Approach for the Development of Profile HMMs

#### 3.3.1. Motivation

Our results, shown above, suggest that neither full-length protein sequences nor short unselected sequences can be universally applied to detect viral taxonomic groups, especially considering that divergence rates across these groups can be very dissimilar. Hence, the development of a more refined approach for the automatic identification of conserved and discriminative regions of an MSA, using non-subjective rational approaches, would be of great relevance. This challenge prompted us to develop TABAJARA, a Tool for Alignment Block Analysis Joining Appropriate Rational Approaches.

#### 3.3.2. Workflow of the Program

**Figure 3:**
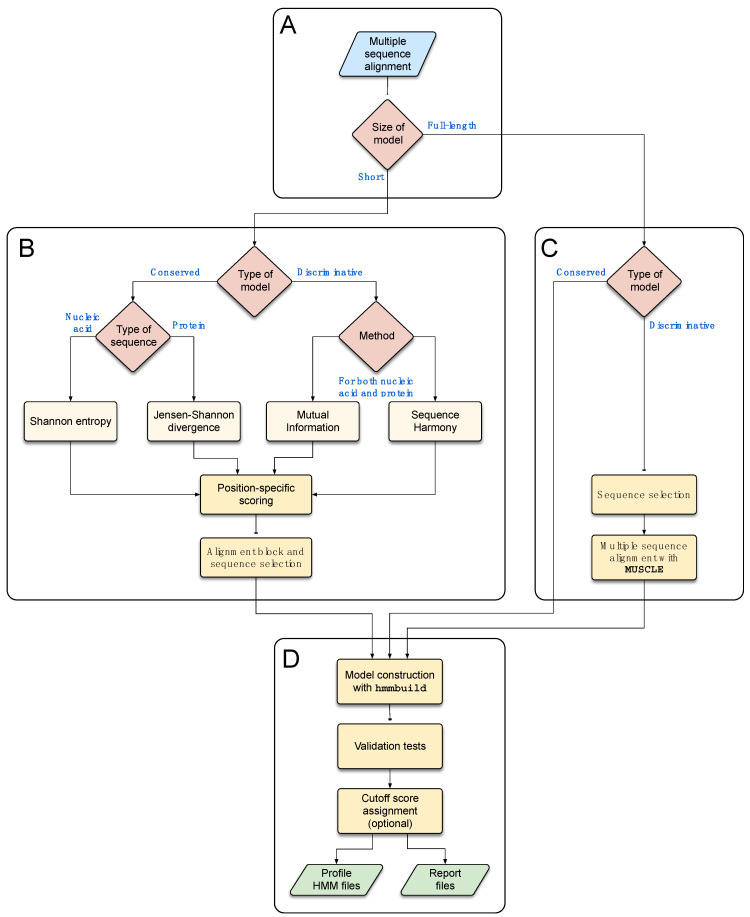
Workflow of profile HMM construction. A multiple sequence alignment is used as an input training set to construct models from short alignment blocks or full-length sequences (**A**). TABAJARA can be executed in conservation and discrimination modes. For short models (**B**), the program calculates position-specific scores using different metrics, according to the nature of the sequences (nucleotide or protein) and execution mode. Top-scoring regions are selected using a sliding window approach and extracted. Full-length models can also be constructed in both execution modes (**C**). For discrimination mode, group-specific sequences are selected and realigned with MUSCLE. In the case of conservation mode, the original alignment is used for model construction. At the final step (**D**), short or full-length alignments are used to construct profile HMMs using hmmbuild. All models are submitted to a validation procedure involving sensitivity and specificity tests, according to user-defined criteria, and automatic cutoff scores can be optionally assigned. Models fulfilling the quality criteria and report files are stored. See main text for details.

The program’s workflow (Figure 3) begins with an MSA file containing homologous sequences that are either taxonomically or functionally related and represent the diversity of a given group, thus constituting a training set for model development. Both nucleotide and protein sequence data can be used in the MSA. If specified by the user, the program can detect identical sequences to eliminate redundancy and remove gap-only columns. TABAJARA can be used for the construction of either profile HMMs derived from full-length sequence MSAs or from short alignment blocks. The program can be executed in two distinct modes, according to the user’s objectives: (1) conservation, to construct profile HMMs able to detect all sequences of the MSA; and (2) discrimination, to build models that can discriminate a specific group of sequences regarding the remaining sequences of the MSA. If the user executes the program with a full-length sequence dataset using the conservation mode, the model is directly constructed from the MSA. When the discrimination mode is selected, TABAJARA extracts only those sequences belonging to the chosen group, realigns them with MUSCLE and executes hmmbuild to construct the profile HMM. For short models, in both execution modes, selected alignment blocks are used to build profile HMMs which in turn reflect particular specificities of the respective regions. In conservation mode, the program uses Shannon entropy [68,69] or Jensen–Shannon divergence [10] to calculate position-specific scores for nucleotide and protein sequence data, respectively. In discrimination mode, the main objective is finding autapomorphic sites that allow discriminating a subgroup of sequences (e.g., DENV sequences) from the other sequences of the training set (e.g., other *Flavivirus* sequences). For this task, TABAJARA uses a combination of two different scoring methods, mutual information (MI—see Section 2.6.3) [9,15] and sequence disharmony (SD—see Section 2.6.4) [13,14], for both nucleotide and protein sequence data. If more than one category (sequence class) of interest is specified, TABAJARA finds discriminative alignment blocks to each category by running multiple iterations. The entire execution process is very fast and does not demand high computational power or a large amount of RAM. Considering a typical MSA, composed of 127 protein sequences, with a sequence length of about 3300 aa residues (*Flavivirus* dataset—Section 2.1.2), the execution time is no longer than a few minutes, even with using a typical notebook running Linux.

#### 3.3.3. Alignment Block Selection

Whatever the scoring method chosen by the user, TABAJARA joins the most informative positions into contiguous regions. The program implements a sliding window approach where the user can define the sliding window size (parameter w). The accepted windows must present a minimum percentage of position scores (parameter p) above a given threshold (parameter t). Contiguous valid windows are then merged into alignment blocks, which in turn are joined to each other, if separated by a distance less than the maximum allowed value (parameter md). Only alignment blocks longer than a minimum pre-defined size (parameter b) are accepted. If a maximum block size is specified (parameter mb), TABAJARA presents the region with the highest score sum within each alignment block. This process can optionally be iterated in the remaining stretches of the larger alignment blocks to select additional regions of the specified size. Selected alignment regions comprising all sequences (conservation mode) or a subset of sequences, restricted to the group of interest (discrimination mode), are then extracted from the MSA and submitted to the following processing steps before profile HMM construction. First, identical sequences are detected and only one representative of each redundant set is maintained. Next, sequences presenting percentages of gap characters higher than a user-defined value are discarded. Gap-only columns are also identified and removed from the alignment block. Finally, TABAJARA only uses blocks composed of a user-defined minimum number of sequences for model construction.

#### 3.3.4. Profile HMM Construction

Selected alignment blocks are used as input for model construction using hmmbuild (HMMER 3 package) [21]. Default hmmbuild parameters are used unless otherwise specified by the user.

#### 3.3.5. Model Validation

After building the profile HMMs, TABAJARA executes a series of validation steps to select only models that fulfill a set of quality criteria. First, models are submitted to similarity searches with hmmsearch against all sequences of the input MSA. TABAJARA then inspects the results and checks whether the models meet the user-defined quality control criteria. In Conservation mode, each model is tested for the ability to detect a minimum overall recall value of sequences of the training set (parameter -pd, default = 80%). In the discrimination mode, the aim is to obtain models specific to sequences of a given category (e.g., group A), able to discriminate them from the remaining sequences of the training set (e.g., group B). In this case, the top hit sequence of the similarity search must be a member of group A, otherwise the model is discarded. If sequences of the group B are also detected, TABAJARA screens the list of hits in decreasing order of the best domain bit score and calculates the proportion between the score of the first positive sequence of group B in relation to the score of the immediately preceding sequence of group A. This number must be equal to or lower than a user-defined value (parameter -pt, default = 80%), otherwise the model is discarded. As a final checkpoint, all models validated at this stage are tested for their ability to recall sequences of the chosen category to meet the category-specific recall value (parameter -pc, default = 80%).

#### 3.3.6. Cutoff Score Assignment

The main application of profile HMMs is their use in similarity searches against datasets of DNA or protein sequences. The high sensitivity of this methodology often comes with a drawback in terms of low specificity. Our preliminary analyses (Figure 1 and Figure 2), using two very distinct viral groups, have shown that cutoff scores corresponding arbitrarily to 80% of the score observed for the last hit of the training set, represent a good compromise between recall and specificity. Hence, we decided to implement a heuristic approach in TABAJARA that automatically assigns custom cutoff scores for every model. When running TABAJARA in conservation mode, the assigned cutoff score follows this criterion. In the case of the discrimination mode, if all training set sequences detected by a model during the validation procedure belong to the chosen category (e.g., group A), TABAJARA assigns a cutoff score based on a user-defined percentage (parameter -pt) of the lowest score observed in the similarity search. When hits corresponding to sequences of group B are also detected, the program screens the list of hits in decreasing order of best domain bit score and calculates the absolute difference between the scores of the first positive sequence of group B (e.g., 30) and the immediately preceding sequence of group A (e.g., 150). A value corresponding to 80% of this difference (in this example 0.8 × (150 − 30) = 96) is added to the score of the top performer sequence of group B (30). Thus, in this case, the cutoff score would be 30 + 96 = 126. The rationale behind such heuristics is to ensure that remote sequences belonging to the chosen category, but presenting a slightly lower score (80%), would still be detected by the model, whereas sequences not belonging to this category would remain classified as negative. Once the appropriate cutoff scores are determined, TABAJARA inserts a corresponding tag in the profile HMM header. The tag (e.g., CUTOFF SCORE 45) itself is neutral to programs of the HMMER package (hmmsearch and nhmmer) but can be specified by using the parameter -T of these programs (e.g., -T 45). Some bioinformatics applications developed by our group, such as GenSeed-HMM [47], HMM-Prospector (https://github.com/gruberlab/hmmprospector—URL accessed on 2 February 2023) [52], and e-Finder (https://github.com/gruberlab/efinder—URL accessed on 2 February 2023) [52] can automatically read this tag in each profile HMM and use its respective value in the parameter -T when invoking hmmsearch for the similarity searches.

### 3.4. Factors Affecting the Performance of Profile HMMs

#### 3.4.1. Parameter Optimization

TABAJARA is highly flexible, allowing the use of a large set of parameters in the construction of profile HMMs. Obligatory parameters include the minimum size of the alignment block, minimum information score threshold, and minimum percentage of meaningful positions per window. To evaluate the impact of these parameters in the performance of the constructed models, we conducted a series of experiments using the *Flavivirus* 127-sequence training set (see Section 2.1.2). We executed the program in discrimination mode to obtain DENV-specific models varying one parameter at a time. All models were evaluated against the *Flavivirus* 6364-sequence test set (Section 2.1.2) by running hmmsearch with the program’s default cutoff (parameter E equal to 10) or using cutoff scores ascribed by TABAJARA. To assess the classification performance of the profile HMMs, we estimated the sensitivity and specificity values for every individual model and then calculated the arithmetic means of each set of multiple models obtained with the corresponding values of the evaluated parameter. In the first experiment, we varied the information score threshold (parameter t) and maintained the other parameters unchanged (Figure S1A). The observed sensitivity of the models was very close to 1.0 irrespective of the value of parameter t, but specificity was only high when using threshold numbers above 0.6. Conversely, TABAJARA failed to generate models for threshold values above 0.7 because no alignment block fulfilled this highly stringent condition. When we used automatically ascribed cutoff scores, the overall performance improved dramatically, with sensitivity and specificity reaching numbers close to 1.0 for all tested values of parameter t. Similar results were also observed when performing the same experiment using ZIKV (Figure S1B) and YFV (Figure S1C).

In a second test, we measured the effect of the minimum percentage of meaningful positions per window (parameter p) (Figure S2A). When using hmmsearch without cutoff scores, the results were very similar to those observed in the former experiment. The constructed models showed very high sensitivity values, but specificity was only close to 1.0 with values of p higher than 70. Conversely, when cutoff scores were employed, the performance improved dramatically, with sensitivity and specificity values reaching numbers close to 1.0 for all tested numbers of parameter p. Values of p above 85 were too stringent and yielded no models. The same behavior was also observed when using ZIKV (Figure S2B) and YFV (Figure S2C). In our experience, the results depicted in Figures S1 and S2 may vary slightly when using datasets of other viral groups but are in general relatively similar (data not shown). Nevertheless, using cutoff scores increase specificity values under different conditions, thus reducing the need of fine-tuning parameters before running the program.

#### 3.4.2. Alignment Block Size

In the experiment depicted in Figure 2, we concluded that the size of the unselected short alignment blocks has a direct influence on the discriminative ability of the resulting profile HMMs. To assess whether this feature has a relevant influence on models constructed from alignment blocks selected by TABAJARA, we set the program to use equal values for minimum and maximum block sizes (equal values for parameters b and mb), as to generate profile HMMs of specifically defined lengths. We built and tested the DENV-specific models using the same training and test datasets described previously. Recall and specificity values were individually computed for all profile HMMs and arithmetic means of these values were calculated for models of the same size (Figure S3A). As can be seen, models of all sizes showed very high recall rates using or not cutoff scores. This result can be ascribed to the fact that polyproteins of distinct *Flavivirus* species show high similarity, ranging from 39.9% to 99.9% (mean of 61.1% ± 12.6), as calculated with the Needle program. In terms of specificity, the use of cutoff scores yielded very high values in models of all sizes. Conversely, without the use of cutoff scores, specificity is high for short models (10-aa) but shows a large decrease with sizes longer than 20 amino acid residues, similarly to what has been observed when using unselected short blocks (Figure 2). This result can be explained since taxon-specific signatures, rich in autapomorphic positions, may be restricted to very short stretches, whereas long sequences may contain increasing amounts of synapomorphies, positions that are conserved across multiple taxa. It is worth mentioning that even though we used a relaxed set of parameters in this experiment, TABAJARA failed to generate models longer than 300 aa. Analogous tests were performed for ZIKV (Figure S3B) and YFV (Figure S3C) using TABAJARA, with similar results being observed. This body of evidence suggests that discriminative models constructed by TABAJARA can be highly specific for different taxa, provided that cutoff scores automatically ascribed by the program are used. Conversely, if cutoff scores are ignored, the user needs to choose a correct set of parameters to select short and highly discriminative alignment blocks for profile HMM construction.

#### 3.4.3. Sample Size of the Training Set

**Figure 4:**
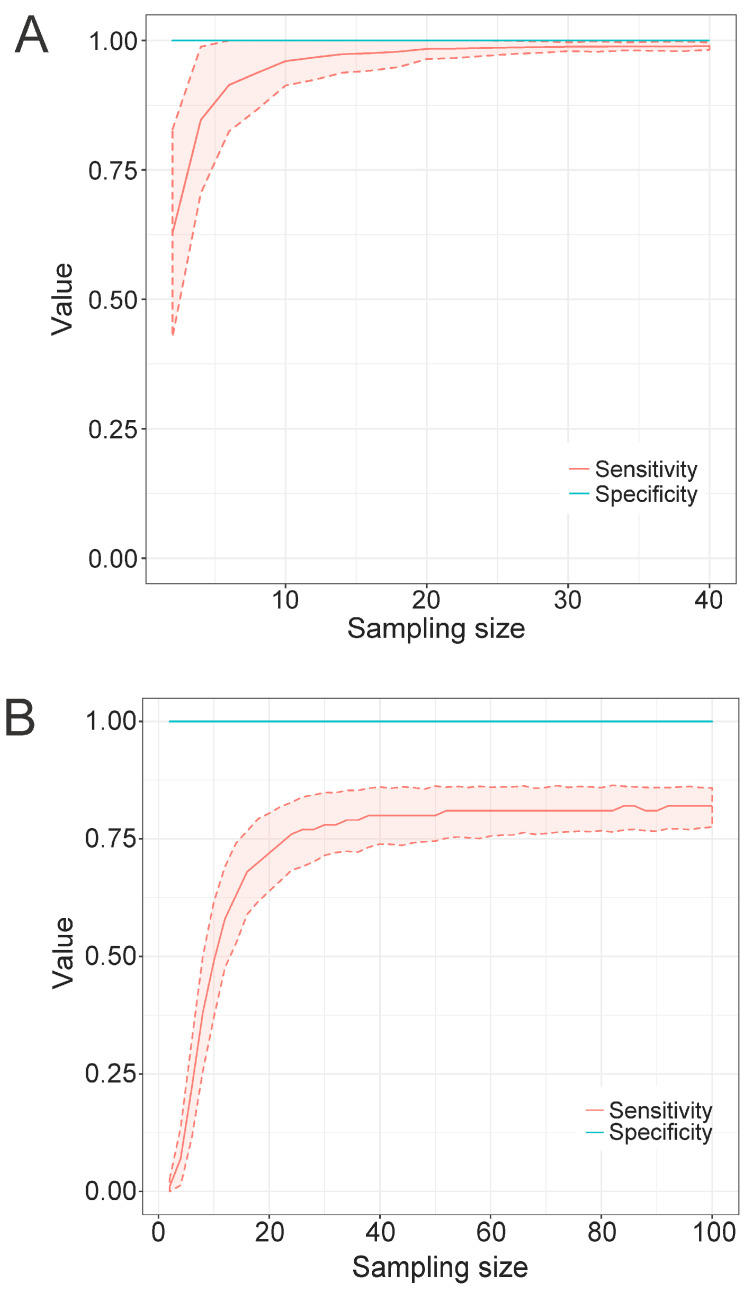
The effect of sequence sampling size on the performance of profile HMMs constructed with TABAJARA. A DENV-specific alignment region (coordinates 10–117), derived from an MSA composed of 6364 *Flavivirus* polyprotein sequences, was selected. A second test was performed with a *Gokushovirinae*-specific alignment region (coordinates 2240–2339), derived from an MSA composed of 1866 *Microviridae* VP1 sequences comprising *Alpavirinae*, *Gokushovirinae* and *Pichovirinae* representatives. Alignment blocks covering the selected regions, composed of an *n* number of randomly sampled taxon-specific sequences, with 1000 pseudoreplicates for each *n* value, were used to construct profile HMMs. All DENV-derived models were tested with hmmsearch program against a bona fide dataset of 6364 *Flavivirus* (**A**) sequences comprising a wide variety of viral species. For *Microviridae* (**B**), profile HMMs were validated against a bona fide dataset of 1866 VP1 sequences, comprising members of three subfamilies of the family. In all tests, the datasets were specifically depleted of the DENV or *Gokushovirinae* sequences used to construct the respective evaluated models. Sensitivity and specificity lines, using cutoff scores ascribed by TABAJARA, represent arithmetic means calculated from the combined results of models generated with the pseudoreplicates of each sampling size. Shaded areas indicate standard deviation values.

In the previous experiments, using a standard training set of 127 *Flavivirus* polyprotein sequences, we tested different program parameters to optimize the construction and performance of the resulting discriminative models. Since all profile HMMs are constructed from an MSA, which is the training set, this set of sequences should consist of a highly representative sample of the diversity found in each population. We then decided to assess how many sequences would be optimally representative of such diversity and be able to yield models displaying high recall and specificity rates. To perform such an evaluation, we ran TABAJARA on the 6364-sequence *Flavivirus* dataset (comprising 3919 dengue and 2445 non-dengue sequences) and found 28 regions discriminative for DENV. For each discriminative region found, the corresponding alignment block was extracted from the full MSA. We used a script to produce random samples composed of *n* DENV sequences without duplicates within each sample. For each *n* value, varying from 2 to 40 sequences, a total of 1000 pseudoreplicates were generated. At the end, all sequence samples were used to construct the respective profile HMMs. To calculate cutoff scores for the models, we used the same heuristics implemented on TABAJARA. Thus, for each model constructed from a training set of *n* DENV sequences, we used this set as the positive control and a dataset of the 2445 non-DENV as the negative control. All models were then submitted to similarity searches with hmmsearch, using the corresponding cutoff scores, against the original 6364-sequence *Flavivirus* dataset depleted of the DENV sequences used to construct the respective tested model. Finally, we calculated the sensitivity and specificity rates of all models and computed the arithmetic means of these values for each set of pseudoreplicates. Figure 4A depicts the results of models obtained from one (coordinates 10–117 of the MSA) of the 28 DENV-specific regions. We obtained a specificity value of 1.0, regardless of the number of sequences used to construct the models. The same result was also observed when testing the other models derived from the remaining 27 DENV-specific regions (data not shown). The set of results indicates that TABAJARA is highly effective to find alignment blocks that yield taxon-specific profile HMMs. Regarding sensitivity, we observed a correlation between the number of sequences used to build the models and the corresponding recall rates. Models constructed with only two sequences present a recall of around 0.6, whereas those built with six sequences show a recall rate of approximately 0.9. The recall curve shows saturation at around 20 sequences, with larger numbers of sequences not yielding significant increments in the sensitivity rate. The analysis of the remaining discriminative regions revealed similar results, with recall curve saturation being observed in the range of 8 to 50 sequences, with a mean value of 21 ± 8.5 and median value of 20 (data not shown). We conclude that in the case of DENV, 20 sequences represent the minimum number required for the construction of a reliable profile HMM. To assess the minimum number of sequences necessary to produce reliable models in a more diverse group of viruses, we repeated the same analysis by running TABAJARA on a dataset of 1866 *Microviridae* VP1 sequences (comprising 501 *Alpavirinae*, 1040 *Gokushovirinae* and 325 *Pichovirinae* sequences). We found a total of three regions discriminative for *Gokushovirinae* sequences. Figure 4B displays the results observed for a region covering the coordinates 2240–2339 of the MSA. Analogously to what was observed for DENV (Figure 4A), a specificity value of 1.0 was obtained for models constructed with all sampling sizes. Conversely, the observed recall rates converged to a value of circa 0.8, much lower than which was observed for DENV. The other *Gokushovirinae*-specific regions also showed comparable results, with recall curve saturation being observed in the range of 38 to 78 sequences, with a mean saturation at recall values around 61.0% ± 17.6 (data not shown). The lower recall obtained for *Gokushovirinae* can be ascribed to the much higher divergence observed among sequences of this subfamily (mean of 59.6% ± 7.5 similarity) than across DENV sequences (mean of 89.7% ± 6.3 similarity), as determined by the Needle program. These results indicate that for highly diverse groups, no single short discriminative region can generate profile HMMs with high recall values.

#### 3.4.4. Combining Multiple Models for Detection

**Figure 5:**
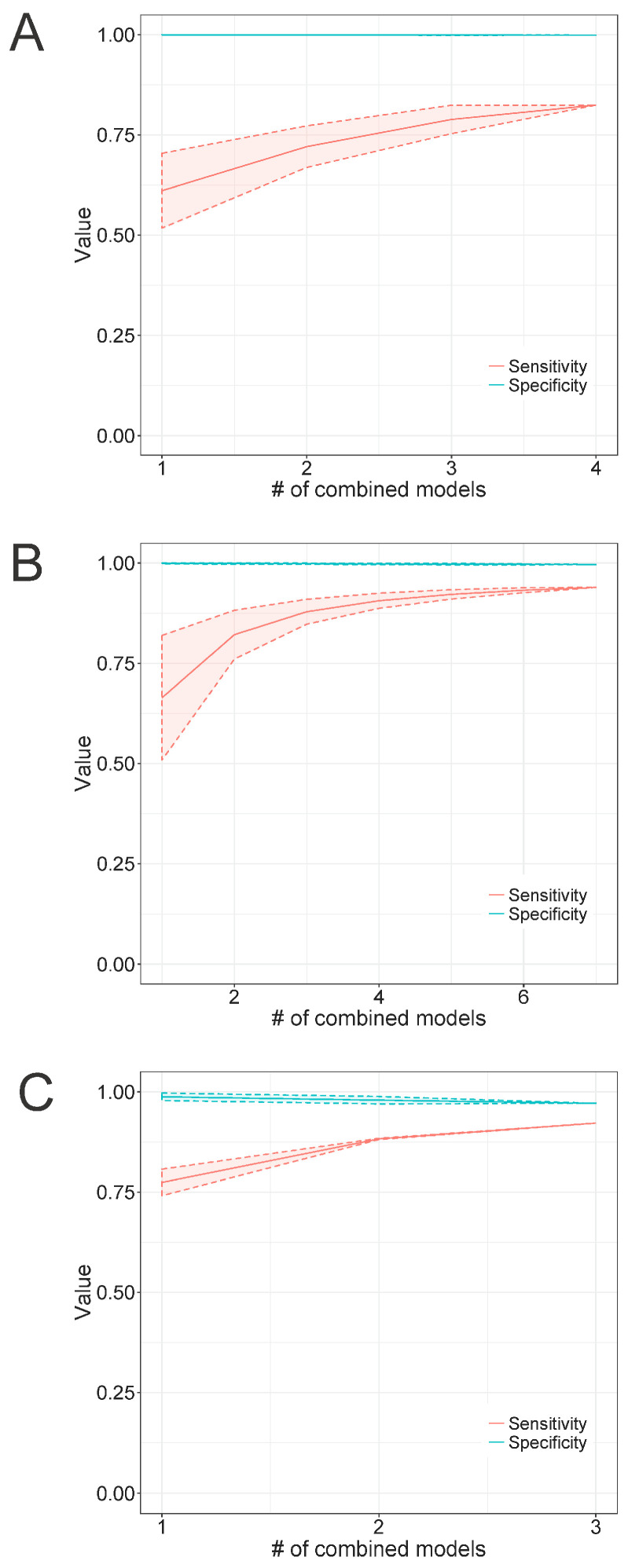
The effect of combining multiple profile HMMs on the ability to detect and discriminate *Microviridae* sequences. TABAJARA was executed in discrimination mode using a training set composed of 113 *Microviridae* VP1 sequences. Models specific for *Alpavirinae* (**A**), *Gokushovirinae* (**B**) and *Pichovirinae* (**C**) subfamilies were tested in similarity searches using hmmsearch program against a bona fide dataset of 1836 *Microviridae* VP1 sequences comprising members of three subfamilies of the family. Tests were performed using multiple combinations of an *n* number of randomly selected models, ranging from one to the total number of models constructed for each subfamily. Sensitivity and specificity lines, using cutoff scores ascribed by TABAJARA, represent arithmetic means calculated from results observed for the multiple combination of models generated for each value of *n*. Shaded areas indicate standard deviation values.

Since we observed in the previous experiment that single models can present low recall values when used for the detection of highly diverse groups, we decided to assess whether the combined use of multiple models would improve the recall rate without substantially impacting the specificity. Thus, we used *Microviridae* as a case study by running TABAJARA for the construction of multiple short models specific to *Alpavirinae*, *Gokushovirinae*, and *Pichovirinae*, using the 113-sequence dataset (see Section 2.1.1) for training. All constructed profile HMMs were submitted to similarity searches using hmmsearch against the 1836-sequence *Microviridae* test set, with the respective cutoff scores of each individual model. For each subfamily, short discriminative models were tested using all possible combinations of *n* models, where *n* ranged from one to the total number of models available for each subfamily. Sensitivity and specificity values were calculated for every random combination of *n* models and arithmetic means were then computed for each set of combined models. Individual models yielded relatively low recall rates, with values around 0.6 for *Alpavirinae* (Figure 5A) and *Gokushovirinae* (Figure 5B) and slightly above 0.75 for *Pichovirinae* (Figure 5C). The combined use of multiple models increased the recall, with the maximum values being observed when using all available models of each subfamily. In all cases, the curve showed a tendency for saturation, with maximum recall values of 0.94 for *Gokushovirinae* and 0.92 for *Pichovirinae*. Conversely, *Alpavirinae* showed a maximum recall value of 0.82, the worst of all subfamilies. This result can be ascribed to the fact that this subfamily shows a mean similarity of 37.0% ± 10.8 across VP1 sequences, whereas *Gokushovirinae* and *Pichovirinae* present mean distance values of 59.6% ± 7.5 and 54.0% ± 6.5, respectively, as determined by the Needle program. This higher diversity of *Alpavirinae*, compared to the other subfamilies, can also be observed in the phylogenetic tree reported by Roux et al. [64], where this subfamily showed the longest branch lengths of all tested *Microviridae* subfamilies. The combined use of models did not cause any noticeable decrease in the observed specificity in *Alpavirinae* (Figure 5A) and *Gokushovirinae* (Figure 5B), with only a slight reduction being observed in *Pichovirinae* (Figure 5C). This body of evidence indicates that finding alignment regions able to discriminate a highly diverse group from its sister taxa while maintaining a high recall is a challenging task. Nevertheless, our results suggest that combining multiple discriminative models can significantly improve overall sensitivity without sacrificing specificity.

#### 3.4.5. Generalization Performance

To evaluate the effect of different training sets on the performance of the resulting profile HMMs and therefore on the robustness of the method, we performed k-fold cross-validations for the *Flavivirus* and *Microviridae* datasets. In the case of the *Flavivirus* genus, we used a dataset composed of 6364 *Flavivirus* polyprotein sequences, comprising 3919 sequences of DENV and 2445 of other *Flavivirus* species. For iteration of the k-fold cross-validation, we constructed the models using TABAJARA in discrimination mode with the following parameters: w 15, t 0.5, p 50, b 20 and mb 60 (see Section 3.3.3 for details). Similarity searches against the test set were performed using or not cutoff scores assigned by TABAJARA. For *Microviridae*, the dataset composed of 1040 *Gokushovirinae* sequences and 826 non-*Gokushovirinae* sequences (comprising *Alpavirinae* and *Pichovirinae* sequences). Since *Microviridae* viral sequences are much more divergent than *Flavivirus*, we used a set of less stringent TABAJARA parameters for model construction: w 12, t 0.3, p 30, b 15 and mb 60. K-fold cross-validation of *Flavivirus* generated a range of 30 to 32 dengue-specific profile HMMs for each iteration, with all iterations showing models with mean recall values of 1.0, whether or not using cutoff scores. All iterations also generated models with specificity values of 1.0 when using cutoff scores. Conversely, specificity decreased dramatically when cutoff scores were not used, falling to mean values varying from 0.34 to 0.44. The k-fold cross-validation of *Microviridae* yielded 4 to 6 profile HMMs per iteration for *Gokushovirinae* (Table S2). The observed mean sensitivity per iteration was 0.85 to 0.92 and 0.98 to 1.0, when using or not using cutoff scores, respectively. This slight decrease in the recall when using cutoff scores was highly compensated in terms of specificity, with all iterations showing a value of 1.0. Conversely, k-fold cross-validation without the use of cutoff scores resulted in much lower specificity values, ranging from 0.50 to 0.63. This set of results shows that model performance is only slightly affected by different samplings of the sequences used to compose the training sets. In fact, the performance is much more influenced by using cutoff scores and the level of intra-group diversity.

### 3.5. Conserved Profile HMMs

Having evaluated the performance of TABAJARA to construct discriminative models, we decided to analyze the ability of the program to generate conserved profile HMMs. We used the standard training and test sets described previously (see Data Sources section) for *Flavivirus* and *Microviridae*. TABAJARA was executed in Conservation mode with the following parameters: w 15, t 0.5, p 50, b 15 and mb 60 (see Section 3.3.3 for details). All models were submitted to similarity searches against the test sets using the hmmsearch program, with and without cutoff scores. We obtained a total of 39 models conserved across *Flavivirus* sequences. These models showed individual recall rates ranging from 0.99 to 1.0 against the 6364-sequence test set, whether or not using cutoff scores. In the case of *Microviridae*, we obtained a total of four profile HMMs, which were tested against the test set of 1836 VP1 sequences, comprising representatives of *Alpavirinae*, *Gokushovirinae* and *Pichovirinae* subfamilies. The individual recall rate of the models varied from 0.74 to 0.85 and from 0.92 to 0.96, when using or not using cutoff scores, respectively (Table S3). Polyprotein sequences of the genus *Flavivirus* are much more conserved than VP1 sequences of the *Microviridae* family, with mean similarity values of 61.1% ± 12.6 and 44.7% ± 13.0, respectively. When normalizing the MSA lengths, we found 39 models for the *Flavivirus* genus, corresponding to 1.02 conserved regions per 100 positions of the MSA. In the case of *Microviridae*, only four models were obtained, with 0.3 conserved regions per 100 positions. Similar to what has been observed for discriminative models (Figure 5), the combined use of conserved models for *Microviridae* improved the recall rates to 0.95 and 0.98, when using or not using cutoff scores, respectively (Table S3).

### 3.6. Profile HMMs for the Discrimination of Transposable Elements

In the preceding sections, we described the development and application of profile HMMs to detect and discriminate different types of viruses. Aiming to provide evidence that this approach can be extended to other biological domains, we decided to build models for the specific identification of casposons, a group of self-synthesizing transposable elements found in archaea and bacteria, that may have originated the CRISPR-Cas adaptive immunity systems [72]. Casposons contain several genes, but those coding for the endonuclease Cas1 and DNA polymerase B are ubiquitous across all elements characterized so far. One of the challenges in detecting new casposons is their differentiation from CRISPRs, which also contain an endonuclease Cas1 gene. Thus, we obtained a dataset of bona fide casposon-derived Cas1 sequences [65] and sequences representing the diversity of CRISPR elements [66]. As previously demonstrated for these Cas1 sequences, [63,65,72] and reanalyzed here (Figure S4), casposons and CRISPRs are monophyletic, showing clearly separated clades. An MSA obtained with MUSCLE was used to run TABAJARA in the discrimination mode to generate models capable of detecting any caposon and discriminating these elements from CRISPRs. In a second execution, we constructed models for the specific detection and discrimination of each of the four casposon families. All models were submitted to similarity searches against casposon and CRISPR sequences. As can be seen in Table S4, we obtained four casposon-specific profile HMMs (labeled with CASPO prefix), that showed recall rates of 100% without the use of cutoff scores and varying from 90.7% to 100% when using cutoff scores. In two cases, CRISPRs were detected only when cutoff scores were ignored. In addition, when used in combination, the four models were able to detect all casposon sequences (not shown). Family-specific models showed 100% recall and specificity rates when using cutoff scores, with two family 4-derived models cross-detecting members of families 2 or 3 when cutoff scores were ignored. Finally, three family-specific models detected CRISPR sequences without using cutoff scores. This set of results shows that TABAJARA can be used to build profile HMMs for other biological applications, allowing to differentiate classes of biological sequences, if they are monophyletic.

## 4. Discussion

### 4.1. A Generic Approach to Construct Profile HMMs

In this paper, we presented the main aspects related to the construction of reliable profile HMMs capable of detecting a set of conserved sequences or discriminating subsets of sequences. First, we observed that models constructed with full-length sequences can detect and discriminate different viral species of the genus *Flavivirus*, provided that proper cutoff scores are used. This ability is less effective for *Microviridae*, a taxonomic family with much higher inter- and intra-group divergence than the genus *Flavivirus*. We also performed a comprehensive set of tests using unselected sequences to better understand the main issues related to this task. The *Flavivirus* genus, composed of relatively similar sequences, is more challenging for obtaining discriminative models. In this case, only profile HMMs constructed from short regions (e.g., 10-aa) are discriminative, unless appropriate cutoff scores are used, whereas high recall rates are usually obtained. Conversely, for the more heterogenous group, the *Microviridae* family, models constructed from unselected short regions (e.g., 10-aa) can be highly discriminative, but due to the high intra-group divergence, such models present low recall rates. Nevertheless, we observed that in groups presenting both low and high intra-group divergence, longer unselected regions corresponded to a lower capacity for discrimination. This result can be ascribed to the incorporation of non-informative positions and/or positions that are shared by other groups of sequences, leading to a loss of specificity. Pearson et al. [73] observed that some regions used for PSSM construction suffer from homologous overextension when very low-identity homologous sequences are added. Such extension, rich in non-specific positions, leads to a decrease in the model specificity. In our case, we observed that longer regions tend to generate less specific models, but the proper use of cutoff scores can at least partially overcome this feature. Thus, long regions tend to generate models that are more sensitive, but at the cost of lower specificity. Conversely, short regions can be highly discriminative, but their recall rates are usually lower. This is a typical paradox of diagnosis, where sensitivity and specificity do not come together in a single assay. To overcome this limitation, TABAJARA was conceived to build models from rationally selected regions, those that are highly informative, avoiding the incorporation of autapomorphic positions for conserved profile HMMs or synapomorphic sites for discriminative models.

TABAJARA scores all positions of an MSA for conservation or discrimination ability and selects the most informative regions, which are rich either in synapomorphies or autapomorphies, respectively. Although TABAJARA does not use any evolutionary model, its approach somewhat resembles molecular phylogeny programs, where information sites are used together under an evolutionary model to reconstruct the relationships of biological sequences. In our experience, TABAJARA succeeds to build highly discriminative models only when the groups to be differentiated are monophyletic, and effective conserved models with high recall rates when the sequences composing the training set are members of the same clade. Future studies may shed light on whether profile HMMs constructed by TABAJARA can be used as phylogenetic markers to specifically detect members of a phylogenetic clade and discriminate them from sequences belonging to other clades. Several tools that identify conserved and discriminative regions have been reported in the literature [2,74,75]. Nevertheless, none of these available applications perform all the steps covered by TABAJARA, including (i) position-specific scoring for either conservation or discrimination using various methods, (ii) identification of the top-scoring regions according to flexible parameterization, (iii) automatic extraction of alignment blocks, (iv) construction of profile HMMs, and (v) model validation.

### 4.2. Cutoff Scores

Several collections of profile HMMs are publicly available, including the more generic Pfam database [24], which is oriented towards protein families, and domain-specific resources, such as VFam [54], pVOGs [53], efam [58], PHROGs [59], and ViPhOGs [60], more oriented to the virology community. With the exception of ViPhOGs and VIRify [61], their accompanying taxonomic classification pipeline, the available models are not provided with cutoff scores, requiring the user to arbitrarily draw the line between significant and non-significant results. Not using cutoff scores at all means that unrelated sequences could be detected. A more common method is to arbitrarily define an e-value cut-off and assume that alignments falling above this canonical value are false positives. While this approach is widely used and straightforward, it can be sometimes misleading. A study using models from the vFAM database [76] employed a cutoff e-value of 1 × 10^−5^ to discriminate viral from human sequences. While the authors found more than 500 viral sequences missed by conventional BLAST-based similarity searches, with an overall rate of 96% of true positive sequences, within the *Mimiviridae* family this rate dropped to 3%. A possible explanation raised by the authors is that these viruses encode genes whose orthologs are also found in cellular organisms. This is an example that the use of arbitrary cutoff values can be risky, especially when surveying viral taxa in metagenomic datasets. Not only many viral proteins resemble prokaryotic and eukaryotic orthologs, but, also, many viral groups share orthologs that must be properly differentiated if a taxonomic classification is required. TABAJARA uses the set of sequences provided in the MSA as a training set to perform validation tests and heuristically determine cutoff scores customized for every profile HMM. The effectiveness of the cutoff scores is obviously dependent on the representativeness of the training set, but it certainly represents an improvement over using arbitrary cutoffs or not using any cutoffs at all.

Some other approaches using cutoff scores to improve the specificity of profile HMMs have been reported in the literature. Pfam [24], a manually curated protein family database assigns score cutoff values to the profile HMMs constructed for all families. To avoid the detection of unrelated sequences by Pfam models, three cutoff scores are assigned to them, which are calculated manually at the time a family is created [77]. When a new family is added to the PFAM database, it is necessary to check for overlapping sequences of this new family with existing ones and cutoff values need to be recalculated. In the case of TABAJARA, if new sequences are added to a particular group, the models must be reconstructed and therefore receive new cutoff score values. However, this process is simple in TABAJARA, as the user only needs to realign the sequences and run the program again to generate the models.

Methodological approaches to classify protein sequences based on profile HMMs have been reported in the literature. Aramaki et al. [78] implemented a method to assign KEGG Orthologs (KOs) that defines an adaptive score threshold that maximizes the F-measure. This method is relatively similar to the one implemented on TABAJARA but, in our case, a sequence of the positive set presenting a bit score lower than any sequence from the negative dataset is classified as negative, thus maximizing model specificity. On the other hand, when cutoff scores are established, we measure the sensitivity of each model, discarding those models that fall below a user-defined minimum value. Chein et al. [79] described a method for the construction of profile HMMs for molecular typing of Enterovirus Type 71 using both nucleotide and amino acid sequences. Bit score-based Z-scores were then calculated for each profile HMM using positive and negative EV71 sequences. The authors concluded that, for EV71, protein-based profile HMMs showed a better performance for molecular typing of enteroviruses. This result cannot be extrapolated to other viruses and applications, since it is highly dependent on the divergence level of each viral group. Cutoff Z-scores were also used to discriminate subtypes of influenza A hemagglutinin (HA) [45]. In both cases, the methods are very specific to the virus model and type of marker chosen by the authors. The method proposed by Gong et al. [45] and the heuristics implemented in TABAJARA make use of the difference between the smallest score obtained for a positive sequence and the largest value obtained for a negative data set. However, instead of using the arithmetic mean between these values, TABAJARA allows the user to define the tolerance level of the models.

Finally, Pagnuco et al. [80] developed the HMMER Cut-off Threshold Tool (HMMERCTTER), a semi-automated, user-supervised procedure that constructs profile HMMs for protein families and assigns cutoff scores to these models. HMMERCTTER employs a user-provided phylogeny to cluster the input sequences, builds profile HMMs for each cluster, and performs similarity searches of the generated models against all input sequences. Profile HMMs that show 100% of sensitivity and specificity are maintained and the lowest score obtained for a positive sequence is defined as the cutoff score. Compared to HMMERCTTER, TABAJARA is more generic and flexible, since it can generate models built with full-length sequences or from the selected informative regions of the MSA. Additionally, TABAJARA uses both nucleotide and protein sequences as input, while HMMERCTTER only works with protein sequences. Regarding cutoff score values, TABAJARA allows the user to define a tolerance level between the positive and negative sequences, whereas HMMERCTTER defines only a fixed value based on the training set. Finally, TABAJARA performs all steps, from region selection to model construction and validation in an unsupervised way. Because the method is generic, TABAJARA can build profile HMMs from virtually any molecular marker of any organism, provided that enough divergence exists between the groups of sequences to be differentiated.

### 4.3. Scope of Applications

In this work, we used viral data to guide the development and application of TABAJARA. Nevertheless, the scope of application of the program is much wider than viral studies, since any monophyletic group of sequences can be discriminated from its sister groups by constructing specific profile HMMs. For instance, we also showed here another application, where profile HMMs derived from endonuclease Cas1 could not only differentiate casposons from CRISPRs with high specificity, but also discriminate the four different families of casposon elements. These models are being successfully used by our group in a comprehensive survey to unravel the diversity of casposons in archaeal and bacterial genomes (unpublished work).

Since TABAJARA identifies the most conserved regions of an MSA, it can be used to discard poorly aligned stretches. Eliminating poorly aligned regions from an MSA has been a widely used approach in molecular phylogeny and is implemented on GBlocks [81], a popular tool for the selection of phylogenetically informative regions. TABAJARA can detect highly conserved regions of an alignment, automatically concatenate these blocks, and store this dataset into a single FASTA file that can be used for phylogenetic analyses. In contrast to GBlocks, TABAJARA is not limited to finding gap-rich regions, as it implements more sophisticated methods based on Jensen–Shannon divergence [10] for protein sequences and Shannon entropy [68,69] for nucleotide sequences. Moreover, because TABAJARA is highly parameterized, a much finer tuning of the conservation level can be attained.

Finding conserved and discriminative regions among related proteins can potentially be used to identify domains associated to specific functions. When applied to diagnostics, protein regions that are discriminative for one or more taxa is a first step to determine potential peptide candidates to compose serological assays. Lee et al. [82] used a similar concept to find *diagnostic sites* in the E glycoprotein and NS1 non-structural protein of ZIKV. The most promising peptide candidates were submitted to further bioinformatic analyses, and the selected molecules were successfully used to discriminate sera from ZIKV-positive patients from other flaviviruses in ELISA assays. Using TABAJARA, a similar approach could be easily extended to many other viral and non-viral pathogens to select potential candidates to compose serological diagnostic assays.

### 4.4. Integration with Other Tools

Profile HMMs produced by TABAJARA can be used with a variety of programs. Our group had previously developed GenSeed-HMM [47], a tool for targeted progressive assembly using profile HMMs as seeds. In addition to TABAJARA, we also provide HMM-Prospector (https://github.com/gruberlab/hmmprospector—URL accessed on 2 February 2023), a companion tool that performs automatic six-frame translation of nucleotide sequences and uses single or multiple profile HMMs to interrogate assembled or unassembled genomic and metagenomic datasets, generating qualitative and quantitative reports. Finally, profile HMMs can also be used for the detection of multigene regions such as proviruses, transposable elements and operons using e-Finder (available at https://github.com/gruberlab/efinder—URL accessed on 2 February 2023). All these tools can automatically use cutoff scores assigned by TABAJARA. A recent review [52] describes an integrated approach for profile HMM construction in the possible applications of these models.

### 4.5. Profile HMMs for Viral Classification

With so many potential applications, profile HMMs are increasingly being used in many studies [44,45,46,47,48,49,50,51], especially in the domain of virology. The currently available databases of viral profile HMMs are highly biased in terms of taxon representation, with many viral groups not represented at all [34]. These databases use different methods to reduce sequence redundancy and are orthology guided, that is, viral sequence groups are obtained by all-versus-all pairwise alignments, followed by a clustering method. Such approach favors grouping sequences that often share the same function but are not necessarily derived from the same taxon. The ability to discriminate different groups of sequences is not a binary property and is directly affected by a wide spectrum of biological variability. Thus, given a set of biological entities to be separated, appropriate arbitrary lines must be established based on a rational set of criteria [83]. In this direction, profile HMMs constructed by TABAJARA represent a major improvement over currently available repositories, since all models are provided with cutoff scores. Such profile HMMs allow to perform similarity searches with much higher specificity than using arbitrary thresholds as is usually reported in viral surveys. Hence, while some of these repositories provide very comprehensive sets of viral data, the associated profile HMMs must be used with caution for viral classification.

Virus classification is a very controversial and dynamic subject. The official virus classification provided by the International Committee on Taxonomy of Viruses (ICTV) [84] until recent years was mostly based on morphological features, viral genome composition and replication mechanism (Baltimore classification) [85], and virus–host relationships. Viral taxonomy has progressively incorporated molecular features [86] among the set of classification criteria. Several approaches have been described for the large-scale classification of viral groups based on genomic data relationships, with very promising results for the development of a wide viral taxonomy scheme based on genomic data [83,86]. It is becoming clear that new repositories of viral profile HMMs are needed to provide a straightforward way to classify current and emergent viruses. In this direction, we have released an experimental version of the Viral Minion Database, a collection of viral profile HMMs (http://www.bioinfovir.icb.usp.br/miniondb/—URL accessed on 2 February 2023) [52] covering prokaryotic and eukaryotic viruses. We provide profile HMMs derived from either short-region or full-length protein sequences. In addition, HMM-Prospector, the companion similarity searching tool, normalizes the cutoff scores according to the length of the target sequences, allowing the screening of both assembled and unassembled sequencing data. We envisage that such taxonomically defined models might be incorporated in an automated pipeline to constitute a reliable viral detection and classification system for metagenomic data, as well as to identifying emerging viruses. Finally, the methodology developed in this work is generic and may be applied to any other biological domain where a sequence class must be specifically detected or differentiated from other classes.

## Data Availability

TABAJARA (https://github.com/gruberlab/TABAJARA—URL accessed on 2 February 2023) and HMM-Prospector (https://github.com/gruberlab/hmmprospector—URL accessed on 2 February 2023) are open-source programs available for download in the GitHub repository under the terms of the GNU General Public License version 3. The programs are fully documented, and tutorials are provided. Datasets used throughout this work, including protein sequences, multiple sequence alignments and constructed profile HMMs are publicly available on Zenodo (https://doi.org/10.5281/zenodo.7490325—URL accessed on 2 February 2023).

## Supplementary Materials

The following are available online at https://www.mdpi.com/article/10.3390/v15020519/s1. Figure S1: The effect of the information score threshold (parameter t) on the performance of profile HMMs constructed with TABAJARA, Figure S2: The effect of the minimum percentage of meaningful positions per window (parameter p) on the performance of profile HMMs constructed with TABAJARA, Figure S3: The effect of the alignment block size on the performance of profile HMMs constructed with TABAJARA. Figure S4: Maximum likelihood tree using full-length amino acid sequences of Cas1 protein sequences from bona fide casposon and CRISPR elements, Table S1: Performance of profile HMMs constructed with full-length VP1 protein sequences for the detection and discrimination of *Microviridae*, Table S2: K-fold cross-validation of profile HMMs constructed with TABAJARA in Discrimination mode for sequences of the *Gokushovirinae* subfamily, Table S3: Sensitivity of conserved profile HMMs for the detection of *Microviridae* sequences, Table S4: Performance of profile HMMs constructed with full-length endonuclease Cas1 sequences for the detection and discrimination of Cas1 sequences of casposons and CRISPR elements.
