# Supplementary material for: Rational Design of Profile HMMs for Sensitive and Specific Sequence Detection with Case Studies Applied to Viruses, Bacteriophages, and Casposons
Source: Viruses. 2023 Feb 13;15(2):519. doi: 10.3390/v15020519 (PMC9966878; doi:10.3390/v15020519)
Supplement: Supplementary file 1 [file viruses-15-00519-s001.zip › Oliveira et al. - Supplementary Material - Viruses final version.pdf]

SUPPLEMENTARY MATERIAL

Rational design of profile HMMs for sensitive and specific sequence detection with case studies applied to viruses, bacteriophages, and casposons

Liliane S. Oliveira [1], Alejandro Reyes [2,3], Bas E. Dutilh [4,5,6] and Arthur Gruber [1,6*]

[1] Department of Parasitology, Instituto de Ciências Biomédicas, Universidade de São Paulo, São Paulo, SP, 05508-000, Brazil; argruber@usp.br
[2] Max Planck Tandem Group in Computational Biology, Department of Biological Sciences, Universidad de los Andes, Bogotá, Colombia; a.reyes@uniandes.edu.co
[3] The Edison Family Center for Genome Sciences and Systems Biology, Washington University School of Medicine, Saint Louis MO, 63108
[4] Institute of Biodiversity, Faculty of Biological Sciences, Cluster of Excellence Balance of the Microverse, Friedrich-Schiller-University Jena, Jena, 07743, Germany; bedutilh@gmail.com
[5] Theoretical Biology and Bioinformatics, Science for Life, Utrecht University, Padualaan 8, 3584 CH, Utrecht, The Netherlands
[6] European Virus Bioinformatics Center, Leutragraben 1, Jena, 07743, Germany
* Correspondence: argruber@usp.br (AG); Tel. +55 11 3091 7274

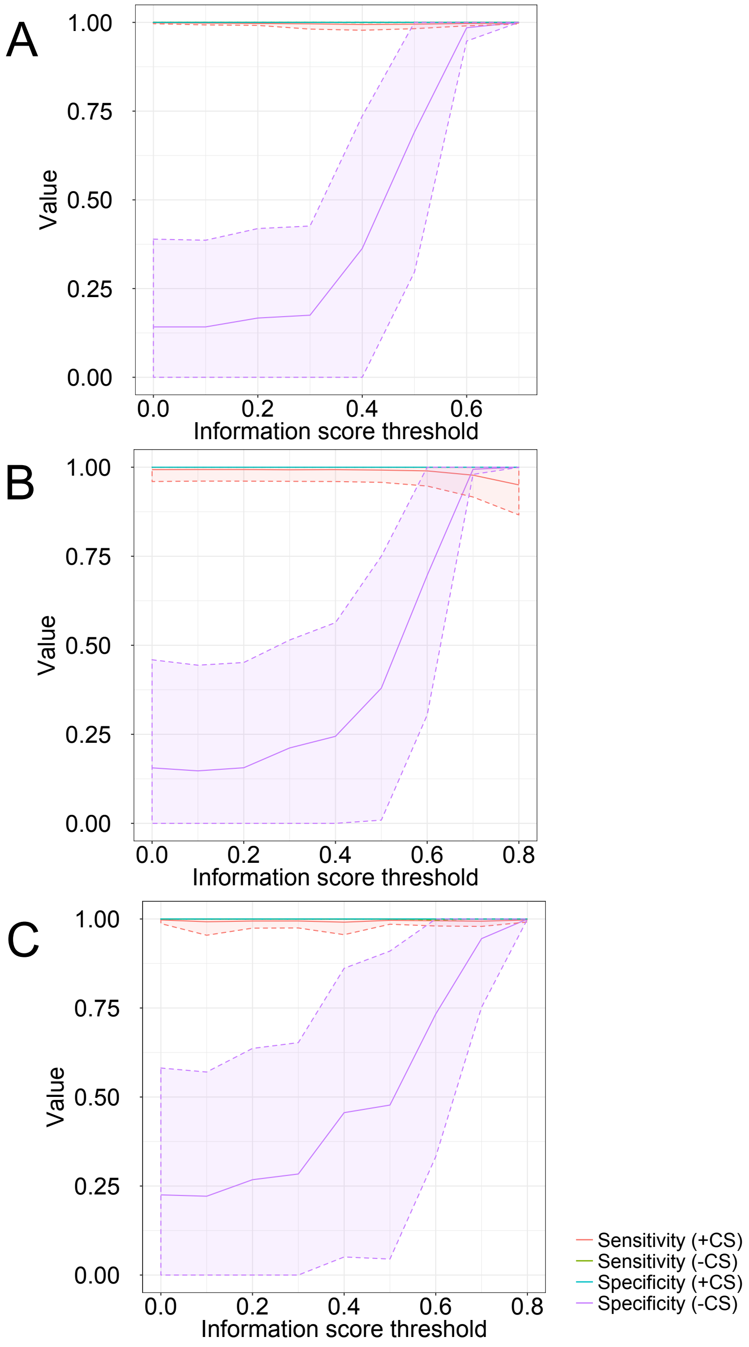

Figure S1. The effect of the information score threshold (parameter `t`) on the performance of profile HMMs constructed with `TABAJARA`. `TABAJARA` was executed in Discrimination mode to generate short models using a training set composed of 127 *Flavivirus* polyprotein sequences. Alignment blocks specific for dengue virus (A), Zika virus (B) and yellow fever virus (C) were selected using a 15-position sliding window, 50% of meaningful positions per window, a block size in the range of 15 to 60, and varying values of the parameter `t`. All models were tested in similarity searches using `hmmsearch` program against a *bona fide* dataset composed of 6,364 *Flavivirus* polyprotein sequences. Sensitivity and specificity lines, using (+CS) or not (-CS) cutoff scores ascribed by `TABAJARA`, represent arithmetic means calculated from the combined results of the multiple models generated for each value of `t`. Shaded areas indicate standard deviation values.

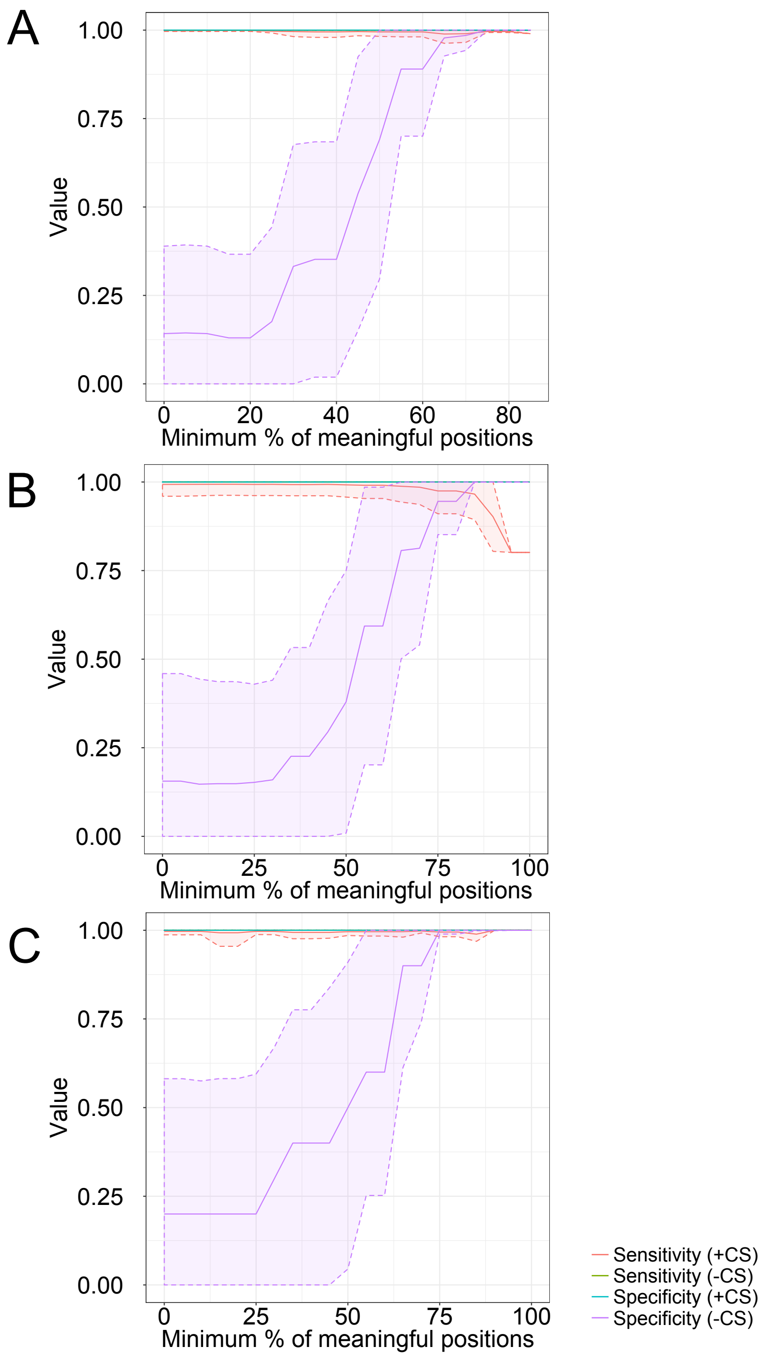

Figure S2. The effect of the minimum percentage of meaningful positions per window (parameter `p`) on the performance of profile HMMs constructed with `TABAJARA`. `TABAJARA` was executed in Discrimination mode to generate short models using a training set composed of 127 Flavivirus polyprotein sequences. Alignment blocks specific for dengue virus (A), Zika virus (B) and yellow fever virus (C) were selected using a 15-position sliding window, an information score threshold of 0.5, a block size in the range of 15 to 60, and varying values of the parameter `p`. All models were tested in similarity searches using `hmmsearch` program against a *bona fide* dataset composed of 6,364 *Flavivirus* polyprotein sequences. Sensitivity and specificity lines, using (+CS) or not (-CS) cutoff scores ascribed by `TABAJARA`, represent arithmetic means calculated from the combined results of the multiple models generated for each value of `p`. Shaded areas indicate standard deviation values.

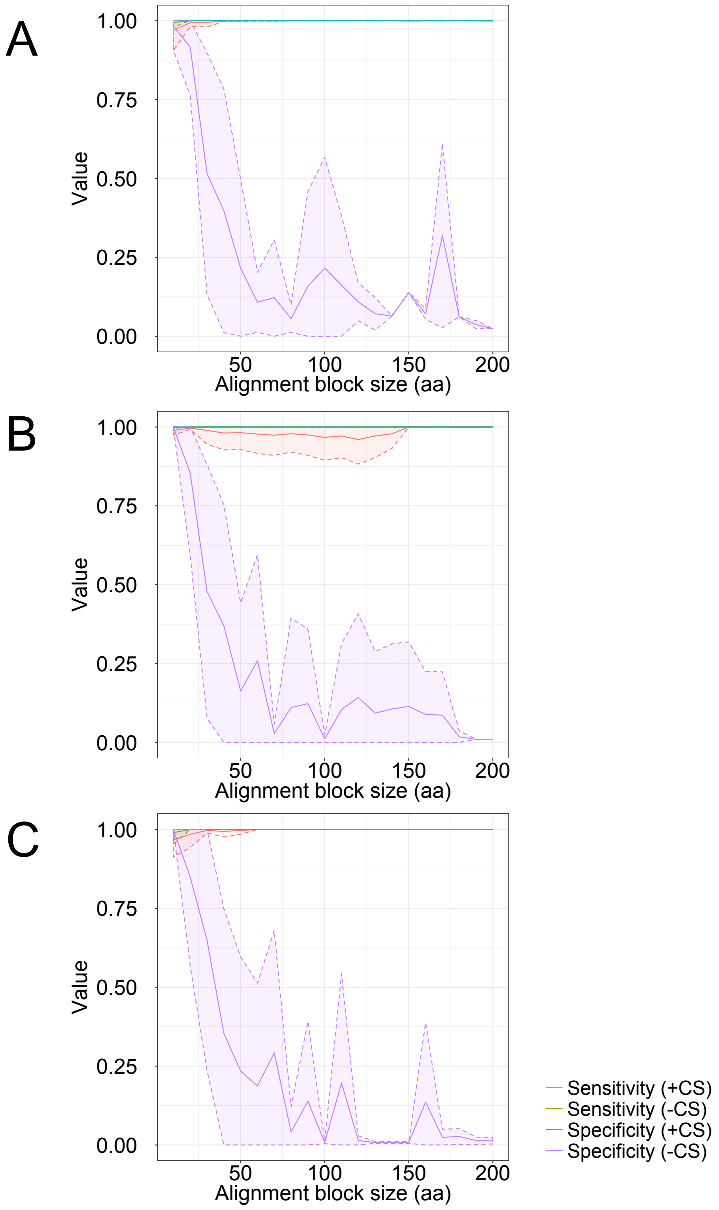

Figure S3. The effect of the alignment block size on the performance of profile HMMs constructed with `TABAJARA`. `TABAJARA` was executed in Discrimination mode to generate short models using a training set composed of 127 *Flavivirus* polyprotein sequences. Alignment blocks of fixed sizes, starting from a minimum length of 10 aa, and varying in increments of 10 aa each, were selected using a 10-position sliding window, an information score threshold of 0.4 and 40% of meaningful positions per window to build profile HMMs specific for dengue virus (A), Zika virus (B) and yellow fever virus (C). All models were tested in similarity searches using `hmmsearch` program against a *bona fide* dataset composed of 6,364 *Flavivirus* polyprotein sequences. Sensitivity and specificity lines, using (+CS) or not (-CS) cutoff scores ascribed by `TABAJARA`, represent arithmetic means calculated from the combined results of the multiple models generated for each alignment block size (defined by equal values of parameters `b` and `mb`). Shaded areas indicate standard deviation values. For the sake of consistency of the plots, alignment blocks longer than 200 positions, presenting specificity values below 0.025 are not shown.

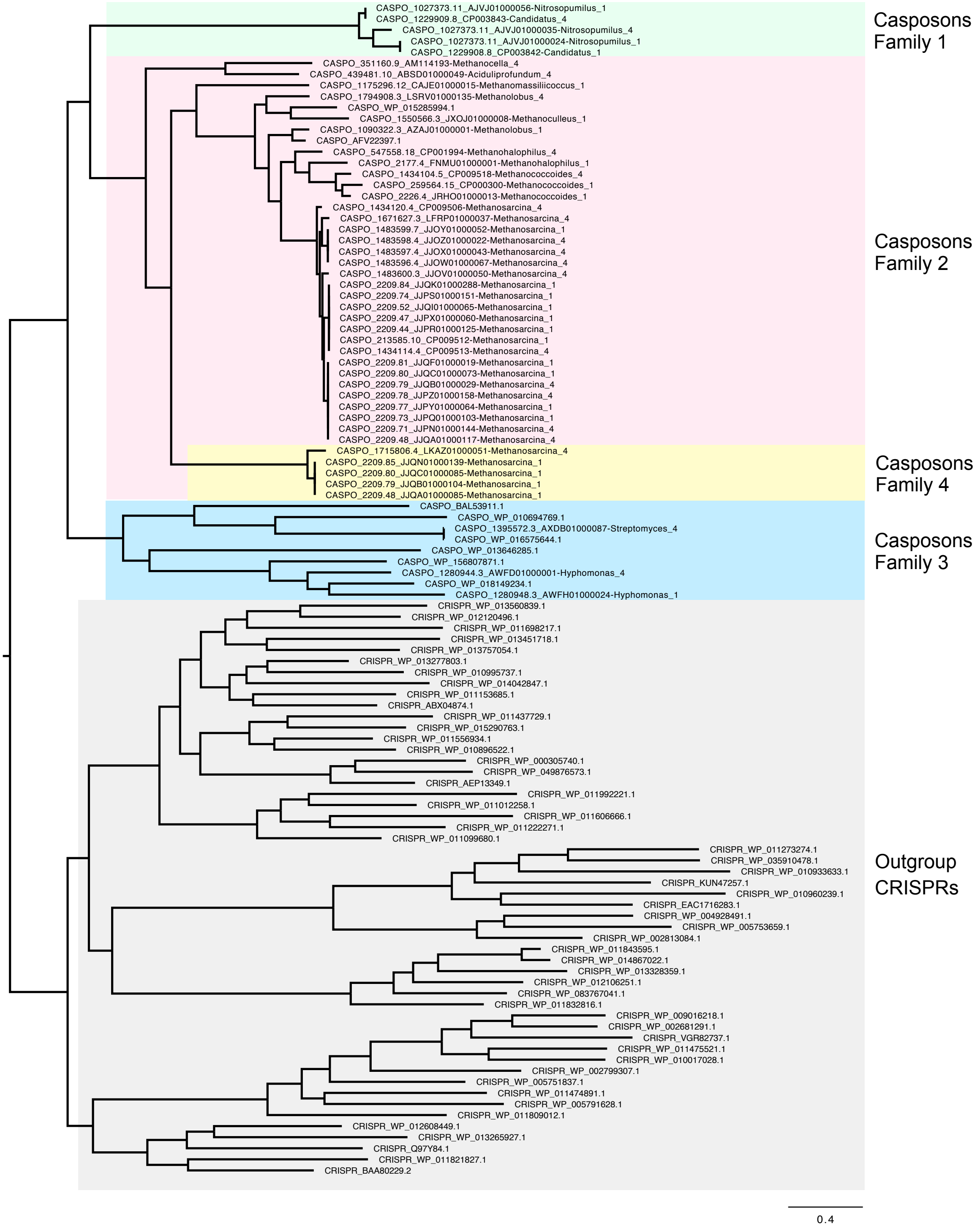

Figure S4. Phylogenetic tree inferred by maximum likelihood using full-length amino acid sequences of Cas1 protein sequences from *bona fide* casposon and CRISPR elements. Phylogenetic reconstruction was performed with `FastTree` using the WAG model of amino acid evolution and a discrete gamma estimation of 20 categories of evolutionary rates across sites. Node support values are shown. The tree was rooted on the node separating casposons and CRISPRs. Colored clades correspond to CRISPRs (grey), and Family 1 (green), Family 2 (red), Family 3 (blue), and Family 4 (yellow) of casposons. Modified and reanalyzed from Krupovic *et al.* 2016 (60).

Table S1 – Performance of profile HMMs constructed with full-length VP1 protein sequences for the detection and discrimination of *Microviridae*. `TABAJARA` was executed in Discrimination mode to generate full-length models using an MSA training set composed of 83 *Microviridae* VP1 sequences, including *Alpavirinae*, *Gokushovirinae* and *Pichovirinae* representatives. All models were tested in similarity searches using `hmmsearch` program against a *bona fide* dataset of 1,866 *Microviridae* VP1 sequences comprising members of three subfamilies of the family. Sensitivity and specificity were comparatively evaluated without cutoff scores, with the use of arbitrary values corresponding to 70%, 80% and 90% of the alignment score observed for the lowest hit of the training set and, finally, with cutoff scores assigned by `TABAJARA`'s heuristics.

Cutoff scores	*Alpavirinae*		*Gokushovirinae*		*Pichovirinae*	
	Sensitivity	Specificity	Sensitivity	Specificity	Sensitivity	Specificity
No cutoff	1.00	0.00	1.00	0.00	1.00	0.00
70%	0.81	0.95	0.98	0.93	1.00	0.34
80%	0.71	0.99	0.97	1.00	0.99	0.38
90%	0.67	1.00	0.92	1.00	0.98	0.57
`TABAJARA`	0.64	1.00	0.87	1.00	0.75	1.00

Table S*2* – K-fold cross-validation of profile HMMs constructed with `TABAJARA` in Discrimination mode for sequences of the *Gokushovirinae* subfamily. A dataset of 1,866 VP1 sequences of *Microviridae*, comprising 1,040 sequences of *Gokushovirinae* and 826 sequences from other subfamilies of *Microviridae* (*Alpavirinae* and *Pichovirinae*), was submitted to a k-fold cross-validation using 10 iterations. For each iteration, sensitivity and specificity values were calculated for individual models and the corresponding arithmetic means of sensitivity and specificity were determined. See main text for details.

Iteration	# of models	With cutoff scores		Without cutoff scores	
		Mean sensitivity	Mean specificity	Mean sensitivity	Mean specificity
1	5	0.88	1.00	0.99	0.60
2	5	0.89	1.00	0.99	0.61
3	5	0.85	1.00	0.98	0.59
4	4	0.90	1.00	0.99	0.60
5	6	0.88	1.00	0.99	0.52
6	5	0.90	1.00	0.99	0.57
7	5	0.85	1.00	0.99	0.52
8	5	0.90	1.00	0.99	0.50
9	5	0.87	1.00	1.00	0.60
10	5	0.92	1.00	1.00	0.63

Table S3 – Sensitivity of conserved profile HMMs for the detection of *Microviridae* sequences. `TABAJARA` was executed in Conservation mode using a training set composed of 113 *Microviridae* VP1 sequences. All models were tested in similarity searches using `hmmsearch` program, with or without cutoff scores ascribed by `TABAJARA`, against a *bona fide* dataset of 1,836 *Microviridae* VP1 sequences comprising *Alpavirinae*, *Gokushovirinae* and *Pichovirinae* representatives.

Profile HMM*	With cutoff scores		Without cutoff scores	
	# of detected sequences	Sensitivity	# of detected sequences	Sensitivity
553-572	1402	0.76	1686	0.92
990-1013	1361	0.74	1738	0.95
1038-1055	1497	0.82	1706	0.93
1127-1146	1560	0.85	1753	0.96
Combined models	1735	0.95	1806	0.98

*The names refer to the coordinates of the alignment blocks in the MSA of the training set.

Table S4 – Performance of profile HMMs constructed with full-length endonuclease Cas1 sequences for the detection and discrimination of Cas1 sequences of casposons and CRISPR elements. `TABAJARA` was executed in Discrimination mode using a *bona fide* dataset training set composed of 54 Cas1 protein sequences of casposons, including representatives of families 1 to 4, and 52 sequences of CRISPRs. All models were tested in similarity searches using `hmmsearch` program using (CS) or not (No CS) cutoff scores ascribed by `TABAJARA`.

Profile HMM*	# Detected/Total (Sensitivity %)											
	Fam 1		Fam 2		Fam 3		Fam 4		All casposons		CRISPR	
	CS	No CS	CS	No CS	CS	No CS	CS	No CS	CS	No CS	CS	No CS
CASPO_1_260-303	5/5 (100)	5/5 (100)	33/35 (94.3)	35/35 (100)	7/9 (77.8)	9/9 (100)	5/5 (100)	5/5 (100)	50/54 (92.6)	54/54 (100)	0/52 (0.0)	0/52 (0.0)
CASPO_4_454-480	5/5 (100)	5/5 (100)	35/35 (100)	35/35 (100)	8/9 (88.9)	9/9 (100)	5/5 (100)	5/5 (100)	53/54 (98.1)	54/54 (100)	0/52 (0.0)	11/52 (21.2)
CASPO_5_493-538	5/5 (100)	5/5 (100)	35/35 (100)	35/35 (100)	9/9 (100)	9/9 (100)	0/5 (0.0)	5/5 (100)	49/54 (90.7)	54/54 (100)	0/52 (0.0)	2/52 (3.8)
CASPO_6_603-667	5/5 (100)	5/5 (100)	35/35 (100)	35/35 (100)	9/9 (100)	4/9 (44.4)	5/5 (100)	5/5 (100)	54/54 (100)	49/54 (100)	0/52 (0.0)	0/52 (0.0)
Fam1_1_665-828	5/5 (100)	5/5 (100)	0/35 (0.0)	0/35 (0.0)	0/9 (0.0)	0/9 (0.0)	0/5 (0.0)	0/5 (0.0)	5	5	0/52 (0.0)	0/52 (0.0)
Fam2_2_853-901	0/5 (0.0)	0/5 (0.0)	35/35 (100)	35/35 (100)	0/9 (0.0)	0/9 (0.0)	0/5 (0.0)	0/5 (0.0)	35	35	0/52 (0.0)	0/52 (0.0)
Fam3_3_479-524	0/5 (0.0)	0/5 (0.0)	0/35 (0.0)	0/35 (0.0)	9/9 (100)	9/9 (100)	0/5 (0.0)	0/5 (0.0)	9	9	0/52 (0.0)	0/52 (0.0)
Fam3_5_593-632	0/5 (0.0)	0/5 (0.0)	0/35 (0.0)	0/35 (0.0)	9/9 (100)	9/9 (100)	0/5 (0.0)	0/5 (0.0)	9	9	0/52 (0.0)	1/52 (1.9)
Fam4_1_231-270	0/5 (0.0)	0/5 (0.0)	0/35 (0.0)	1/35 (2.9)	0/9 (0.0)	1/9 (11.1)	5/5 (100)	5/5 (100)	5	7	0/52 (0.0)	0/52 (0.0)
Fam4_2_404-462	0/5 (0.0)	0/5 (0.0)	0/35 (0.0)	0/35 (0.0)	0/9 (0.0)	0/9 (0.0)	5/5 (100)	5/5 (100)	5	5	0/52 (0.0)	0/52 (0.0)
Fam4_3_478-538	0/5 (0.0)	5/5 (100)	0/35 (0.0)	27/35 (77.1)	0/9 (0.0)	0/9 (0.0)	5/5 (100)	5/5 (100)	5	37	0/52 (0.0)	0/52 (0.0)
Fam4_4_607-646	0/5 (0.0)	0/5 (0.0)	0/35 (0.0)	0/35 (0.0)	0/9 (0.0)	0/9 (0.0)	5/5 (100)	5/5 (100)	5	5	0/52 (0.0)	0/52 (0.0)
